# Genome‐wide identification and expression analysis reveals the drought‐response MAPK genes in peanut (*Arachis hypogaea* L.)

**DOI:** 10.1002/tpg2.70166

**Published:** 2025-12-22

**Authors:** Jie Zhang, Qingying Meng, Alvaro Sanz‐Saez, Charles Chen

**Affiliations:** ^1^ Department of Crop, Soil and Environmental Sciences Auburn University Auburn Alabama USA; ^2^ Huazhong Agricultural University Wuhan China

## Abstract

Peanut (*Arachis hypogaea* L.) is one of the most important oilseed and food crops, and the drought stress remains the primary adverse environmental factor limiting its growth and productivity. Mitogen‐activated protein kinase (MAPK) cascades play crucial roles in various signal transduction pathways, affecting a wide range of physiological processes and drought stress responses in plants; however, the systematic analysis of the MAPK gene family in peanuts remains unexplored. In this study, we identified 30, 16, and 15 MAPK genes in *A. hypogaea*, *Arachis duranensis*, and *Arachis ipaensis*, respectively. RNA‐sequencing analysis in drought‐tolerant and drought‐susceptible genotypes revealed that *Ah_At_MAPK4* and *Ah_Bt_MAPK4* were significantly upregulated under drought stress conditions, with substantially higher induction in drought‐tolerant genotypes compared to drought‐susceptible ones. Weighted gene co‐expression network analysis further identified a drought‐responsive turquoise module highly correlated with drought tolerance traits, and both *Ah_At_MAPK4* and *Ah_Bt_MAPK4* were identified as core regulatory components within this module. Hub gene analysis revealed these MAPKs co‐express with calmodulin‐binding proteins, implicating calcium signaling in drought adaptation. Three‐dimensional structural modeling confirmed both proteins possess canonical bilobed kinase architecture with properly positioned Thr‐Glu‐Tyr motifs and intact catalytic machinery. This genome‐to‐structure analysis identifies *Ah_At_MAPK4* and *Ah_Bt_MAPK4* as key components in drought‐responsive networks and provides molecular targets for enhancing drought resilience in peanut breeding.

## Core Ideas

- A total of 61 mitogen‐activated protein kinase (MAPK) genes were identified: 30 in cultivated peanut (*Arachis hypogaea*), 16 in *Arachis duranensis*, and 15 in *Arachis ipaensis*.
- MAPK genes were classified into four conserved groups (A–D) based on Thr‐Glu‐Tyr (TEY)/Thr‐Asp‐Tyr (TDY) motifs, revealing evolutionary conservation with *Arabidopsis* orthologs.
- Ah_At_MAPK4 and Ah_Bt_MAPK4 showed significantly higher drought‐induced upregulation in drought‐tolerant genotypes compared to drought‐susceptible genotypes.
- Weighted gene co‐expression network analysis (WGCNA) identified both genes as core components of a drought‐responsive module enriched for calcium signaling and terpenoid biosynthesis pathways.
- AlphaFold structural modeling confirmed canonical kinase architecture with properly positioned active sites, providing structural basis for drought tolerance breeding targets.

## Plain Language Summary

Peanut plants, like many crops, face challenges from drought and other environmental stresses. To survive, they rely on special proteins that help them respond to these threats. One important group of these proteins is called the mitogen‐activated protein kinase (MAPK) family, which helps cells send signals to protect the plant. In this study, we identified and analyzed MAPK genes in three types of peanuts, including both cultivated and wild varieties. We found 30 genes in the cultivated peanut and fewer in the wild types. These genes were grouped based on their similarities, and we discovered that some had more complex structures than others. Most importantly, two specific genes were more active in drought‐tolerant peanuts, especially under dry conditions. This suggests that these genes may help the plants survive drought. Our findings could support future efforts to develop peanut crops that are more resistant to stress, helping ensure better yields in tough environments.

**Abbreviations**
ABA: abscisic acid
DEGs: differentially expressed genes
FPKM: fragments per kilobase of transcript per million mapped reads
GO: gene ontology
HMM: hidden Markov model
KEGG: kyoto encyclopedia of genes and genomes
MAPK: mitogen‐activated protein kinase
PCA: principal component analysis
WGCNA: weighted gene co‐expression network analysis

## 1 INTRODUCTION

Peanut (*A. hypogaea*) is a globally important oilseed and food crop valued for its high nutritional content, including oil, protein, dietary fiber, and vitamins (Arya et al., 2016). As an allotetraploid species (AABB, 2*n* = 4*x* = 40) derived from two wild diploid relatives, *Arachis duranensis* (A‐genome) and *Arachis ipaensis* (B‐genome), cultivated peanut possesses unique genomic complexity. Peanut production faces many challenges, including biotic stresses such as pests and diseases, and abiotic stresses including salinity, heat, and particularly drought. Drought stress remains the primary adverse environmental factor limiting its growth and productivity (Puppala et al., 2023). With climate change intensifying the frequency and severity of drought events in major peanut‐growing regions, understanding the molecular mechanisms underlying drought tolerance has become crucial for developing resilient peanut varieties and ensuring food security.

Plants utilize complicated signaling networks to adapt to complex stress conditions when exposed to changes in their natural environment (Hamel et al., 2006; Krasensky & Jonak, 2012; Nawaz et al., 2023; Yang et al., 2020). In these complicated signaling networks, stress‐activated molecular pathways play an important role in responding to various biotic and abiotic stresses (H. S. Kim et al., 2016; Sewelam et al., 2016; Suzuki et al., 2014; Zhu, 2016). These may include injury from external forces, excessive sodium content, cold conditions, drought, and disease invasion (T. Wang et al., 2022). In previous studies, many gene families have been reported to regulate plant abiotic and biotic stresses. For example, the WRKY superfamily of plant transcription factors is involved in regulating response to various stresses (W. Wu et al., 2024), the MYB family plays an important role in hormone signal transduction and abiotic stress tolerance (Ambawat et al., 2013), and mitogen‐activated protein kinase (MAPK) is associated with abiotic and biotic stress adaptation (Manna et al., 2023). The MAPK family of transcription factors is particularly interesting, since they function within the pathway to transfer external stimuli to the cells, and are also critical in the developmental and physiological processes of plants. The MAPK cascade pathway transmits signals to downstream proteins through phosphorylation (He et al., 2020; M. Zhang & Zhang, 2022). Additionally, MAPK cascades are involved in the regulation of growth and development in plants, acting downstream of receptor‐like kinases and playing a role in processes like cell division and differentiation (J. Xu & Zhang, 2015). MAPKs were reported to be categorized into four groups: A, B, C, and D. Members of A, B, and C contain Thr‐Glu‐Tyr (TEY) motif, while members of group D contain Thr‐Asp‐Tyr (TDY) motif (MAPK Group, 2002). The MAPK signaling pathway is comprised of three sequential protein kinases: MAPK, MAPKK, and MAPKKK. In response to external stimuli, MAPKKKs are activated first, then phosphorylate downstream MAPKKs through two serine/threonine residues in S/T‐X3‐5‐S/T conserved motifs (Taj et al., 2010). Finally, the phosphorylated MAPKKs phosphorylate MAPKs, always on threonine and tyrosine residues in TXY motif, which then may phosphorylate other downstream transcription factors and signaling components (L. Chen, Sun, et al., 2020; Jimenez‐Sanchez et al., 2007). The result of the MAPK cascade pathway is the activation of genes responsible for resistance.

Due to MAPK's key role in stress response and as a highly conserved subfamily of protein kinase (Meng & Zhang, 2013), more research has been carried out to understand the mechanism of its actions in plants, alluding to its importance (Colcombet & Hirt, 2008; Kong et al., 2012; J. Xu & Zhang, 2015; R. Xu et al., 2018). In a recent study, researchers investigated the MAPK gene family within Tartary buckwheat (*Fagopyrum tataricum)*, identifying 16 distinct MAPK genes. Their analysis unveiled distinct groupings distinguished by specific motifs and promoter elements, particularly linked to light response, hormone regulation, and responses to abiotic stress. Notably, the expansion of MAPKs containing the TDY motif was observed, hinting at potential roles in developmental processes and stress resistance mechanisms. A total of 18 *AcMAPKs* were identified in kiwifruit (*Actinidia chinensis* L.) (G. Wang et al., 2018), and the expression profiles demonstrated that *AcMAPKs* were induced or repressed by various biotic and abiotic stresses and hormone treatments, suggesting their potential roles in the biotic and abiotic stress response and various hormone signal transduction pathways. A comprehensive analysis identified 17 *LsMAPKs* in lettuce (*Lactuca sativa* L.), and transgenic experiments reveal the silencing of *LsMAPK4* significantly inhibited the high‐temperature‐accelerated bolting in lettuce, indicating that *LsMPAK4* might be a potential regulator of lettuce bolting (T. Wang et al., 2022). A total of five MAPKK genes and 89 MAPKKK genes were identified in tomato (*Solanum lycopersicum* L.), where the expression level of most of these genes changed significantly under multiple treatments, especially in response to salicylic acid and indole‐3‐acetic acid treatment, implying that these genes might have important roles in the plant hormone networks (J. Wu et al., 2014). Additionally, the structurally non‐canonical MAPKK, *MPKK10.2*, could enhance rice (*Oryza sativa* L.) resistance to *Xanthomonas oryzae* pv. *oryzicola* and increase rice tolerance to drought stress by phosphorylating and activating two MAPKs, *MPK6* and *MPK3* (Ma et al., 2017). In drought stress responses, MAPKs function as critical signaling components that integrate stress perception with adaptive responses. Overexpression of the Nicotiana protein kinase *NPK1* enhanced drought tolerance in transgenic maize (*Zea mays* L.) (Shou et al., 2004), while in cotton (*Gossypium hirsutum* L.), a group C MAP kinase gene, *GhMPK2*, positively regulated both salt and drought tolerance through abscisic acid (ABA)‐dependent signaling pathways (L. Zhang et al., 2018). Comprehensive reviews have established that MAPK signaling pathways work in concert with ABA‐dependent pathways to orchestrate plant abiotic stress responses (Danquah et al., 2014). Overall, the main functional categories of MAPKs include abiotic stress signaling, hormone regulation (notably ABA signaling), developmental processes, and biotic stress response. These findings contribute to a foundational understanding of the MAPK gene family and deepen our understanding of stress responses at the molecular level, laying the groundwork for future functional studies and presenting promising avenues for enhancing crop resilience and productivity through targeted interventions.

Despite the agricultural importance of peanut and its susceptibility to drought stress, systematic characterization of the MAPK gene family in peanut remains limited. Early studies have identified individual peanut MAPK genes through heterologous expression: *AhMPK3*, containing a TEY motif, enhanced defense responses when expressed in tobacco (*Nicotiana tabacum* L.) (Kumar et al., 2009), and *AhMPK6* induced hypersensitive response‐like cell death and upregulated defense‐related transcripts (Kumar & Kirti, 2010). In a transcriptome analysis of leaves under well‐watered and drought‐stress conditions for the drought‐tolerant cultivar L422 of peanut, a total of 1313 significantly expressed genes were identified at three time points throughout the drought stress stage; six vital metabolic pathways, including the “MAPK signaling pathway plant,” were enriched under severe drought stress (Zhao et al., 2021). These findings revealed the MAPK genes play a potential role in environmental stress response. Given the significance of the MAPK gene family in plants, it has been extensively studied and identified across various plant species. However, these studies focused on isolated genes or global transcriptomic patterns rather than comprehensive family‐wide characterization.

In this study, we conducted a genome‐wide identification of MAPK gene family members in peanut species, including two diploid species, *A. duranensis* and *A. ipaensis*, and the tetraploid cultivated species, *A. hypogaea*. A total of 30 *Ah_MAPKs*, 16 *Du_MAPKs*, and 15 *Ip_MAPKs* were identified in *A. hypogaea*, *A. duranensis*, and *A. ipaensis*, respectively. We performed a structural analysis along with an investigation into their potential functions under drought stress. The aim of the study was to provide a comprehensive understanding of MAPK signaling pathways in peanuts, which could contribute to future research on gene cloning, expression, and genetic improvement in peanut breeding.

**Core Ideas**

- A total of 61 mitogen‐activated protein kinase (MAPK) genes were identified: 30 in cultivated peanut (*Arachis hypogaea*), 16 in *Arachis duranensis*, and 15 in *Arachis ipaensis*.
- MAPK genes were classified into four conserved groups (A–D) based on Thr‐Glu‐Tyr (TEY)/Thr‐Asp‐Tyr (TDY) motifs, revealing evolutionary conservation with *Arabidopsis* orthologs.
- Ah_At_MAPK4 and Ah_Bt_MAPK4 showed significantly higher drought‐induced upregulation in drought‐tolerant genotypes compared to drought‐susceptible genotypes.
- Weighted gene co‐expression network analysis (WGCNA) identified both genes as core components of a drought‐responsive module enriched for calcium signaling and terpenoid biosynthesis pathways.
- AlphaFold structural modeling confirmed canonical kinase architecture with properly positioned active sites, providing structural basis for drought tolerance breeding targets.

## 2 METHODS AND MATERIALS

### 2.1 Gene resource

To identify MAPK genes within the peanut species, including the diploid ancestors *A. ipaensis* (aradu.V14167.gnm1) and *A. duranensis* (K30076.gnm2.1GWY), as well as the tetraploid cultivated species *A. hypogaea* (Tifrunner.gnm2.J5K5), the genome and protein sequences were downloaded from PeanutBase (https://www.peanutbase.org/). The MAPK protein sequences of *Arabidopsis* were obtained from TAIR (https://www.arabidopsis.org/). The protein kinase domain (PF00069) was downloaded from the Pfam database (https://www.ebi.ac.uk/interpro/entry/pfam/). For gene functional annotation of *A. hypogaea*, InterProScan v5.55‐88.0 was applied to predict gene ontology (GO) term with parameters “‐goterms ‐iprlookup ‐appl Pfam”and eggnog v2.12 was applied to predict the Kyoto encyclopedia of genes and genomes (KEGG) pathway with default parameters.

### 2.2 Genome‐wide identification of MAPK genes in peanut

First, the *Arabidopsis thaliana* MAPK protein sequences were used as the search seed for a BLASTP (McGinnis & Madden, 2004) homology search against the predicted peanut protein models derived from the whole‐genome data with an *e*‐value of 1 × 10^−5^, a minimum identity of 50%, and a minimum coverage of 50% as a threshold. Then, the HMMER v3 (Mistry et al., 2021) program was used to remove redundant sequences by applying the serine/threonine protein kinase‐like domain (PF00069) as a query for hidden Markov model (HMM) searches with a threshold of 1 × 10^−5^. Finally, the SMART database (http://smart.embl‐heidelberg.de/) was then used to detect potential mobile domains and domain architecture. WoLF PSORT (https://www.genscript.com/wolf‐psort.html) was used to predict the subcellular localization of identified MAPK genes (Horton et al., 2007).

### 2.3 Sequence alignment and phylogenetic analysis

The protein sequences of MAPK genes from peanut and *A. thaliana* were aligned using MUSCLE v3.8.31 (Edgar, 2004). Maximum likelihood inference of phylogenetic relationships was performed using PhyML v3.3 (Guindon et al., 2010) with 1000 bootstrap replicates. The phylogenetic tree was visualized by iTOL (https://itol.embl.de/).

### 2.4 Motifs and gene structure analysis

Motif analysis was performed using the MEME Suite version 5.0.5 (Bailey et al., 2015). The tool was configured to identify conserved motifs within the protein sequences. The search was set to discover a total of 10 motifs, with each motif's width ranging from 6 to 100 amino acids. Based on the gff3 file, the gene structure was analyzed using the TBtools v2.008 (C. Chen, Chen, et al., 2020). Finally, TBtools was utilized to visualize the results of gene structure and conserved motif analysis.

### 2.5 Cis‐regulatory element analysis

To further investigate cis‐regulatory elements in the promoter regions of the identified MAPK genes, we obtained 2000 bp sequences upstream of the start codon for identified MAPK genes, and then these sequences were analyzed by the PlantCARE database (https://bioinformatics.psb.ugent.be/webtools/plantcare/html/). The cis‐acting elements of MAPK genes were identified and further visualized using TBtools software.

### 2.6 Chromosome distribution and collinearity analysis

The genomic locations of the identified MAPK genes were determined using annotation files. The chromosomal positions of the identified MAPK genes were then visualized using the TBtools. To identify syntenic genes, protein sequences were compared by all‐versus‐all BLASTP v2.10.0 with the parameters “‐evalue 10e‐5 ‐outfmt 6 ‐num_alignments 15” (Schaffer et al., 2001). Then the putatively homologous genes identified by BLASTP were analyzed for synteny by the MCScanX package using default settings (Y. Wang et al., 2012). The syntenic genes were displayed with JCVI (https://github.com/tanghaibao/jcvi). The homologous gene pairs were used to calculate the estimation of nonsynonymous (Ka) and synonymous (Ks) values by using KaKs_Calculator v2.0 (D. Wang et al., 2010).

### 2.7 Expression analysis of MAPK genes under drought stress

To investigate the expression patterns of the identified MAPK genes under drought stress conditions, we conducted an RNA‐sequencing (RNA‐seq) analysis. The RNA‐seq data were obtained from a previous transcriptomic study performed in our laboratory on cultivated peanut (*A. hypogaea* L.) subjected to drought stress (PRJNA687542) (X. Wang et al., 2021). Four peanut genotypes were selected based on our previously characterized drought response phenotypes. The drought‐tolerant genotypes (C76‐16 and 587) exhibited higher pod yield under drought stress, lower specific leaf area, cooler canopy temperature, and less severe visual drought stress symptoms compared to the drought‐susceptible genotypes (Tifrunner and 506), which showed opposite trends (Carter, 2015; X. Wang et al., 2021). Plants were grown under rainout shelters at the USDA Agricultural Research Service National Peanut Research Laboratory in Dawson, GA. Two treatments were applied: full irrigation throughout the growing season (irrigated control) and middle‐season drought stress initiated 61 days after planting. Drought stress was progressively applied over 4 weeks, with soil water potential reaching −1050 to −1200 kPa during the second week of treatment. Fully expanded leaves were collected from each genotype at the end of the drought period for RNA extraction. Total RNA was extracted using a modified cetyltrimethylammonium bromide method and purified using a Direct‐Zol RNA MiniPrep Kit. RNA quality was assessed using a NanoDrop ND‐1000 spectrophotometer and an Agilent 2100 Bioanalyzer. A total of 24 cDNA libraries (4 genotypes × 2 treatments × 3 replicates) were constructed and sequenced using an Illumina HiSeq 4000 platform. The RNA‐seq data were trimmed of low‐quality reads and adapter sequences using Fastp v. 0.23.4 with default parameters. Clean reads were mapped to the Tifrunner.gnm2.J5K5 reference genome using HISAT2 v2.1.0 (Bertioli et al., 2019; D. Kim et al., 2015). Only those reads with high mapping quality against the reference genome (‐q 20) were kept. The mapped reads of each sample were assembled using StringTie v2.1.1. The gene expression level was normalized using the fragments per kilobase of transcript per million mapped reads (FPKM) method. Principal component analysis (PCA) was performed on the normalized expression data using log2(count +1) transformed counts after filtering low‐expression genes and retaining those with variance >0.5 across samples. The differentially expressed genes (DEGs) were identified using DESeq2 v 1.36.0 with the threshold of fourfold expression changes | log2(fold‐change) | ≥2 and *p*‐value < 0.05.

### 2.8 Weighted gene co‐expression network analysis

Weighted gene co‐expression network analysis (WGCNA) was performed to identify gene modules and hub genes associated with drought stress responses in peanut. Raw RNA‐seq counts from peanut samples under control and drought conditions were normalized using the regularized‐logarithm transformation (rlog) from the DESeq2 package. The gene set for network construction consisted of all identified DEGs across four genotypes, combined with a curated list of 30 MAPK family genes. A soft‐thresholding power was chosen using the scale‐free topology criterion. An unsigned adjacency matrix was then transformed into a topological overlap matrix. Gene modules were identified using dynamic tree cutting (minModuleSize = 30), and modules with high eigengene similarity were merged using a mergeCutHeight threshold of 0.25. Associations between module eigengenes and key phenotypic traits (drought, tolerant, tolerant × drought, and susceptible × drought) were assessed by Pearson's correlation, and the module showing the highest correlation with the tolerant × drought trait was selected. Hub genes in this module were defined by high module membership and gene significance. A sub‐network of the top 20 hub genes and two key MAPK genes (*Ah_At_MAPK4* and *Ah_Bt_MAPK4*) was visualized in Cytoscape (v3.10.3), with edges weight >0.78.

### 2.9 AlphaFold predicted structures analysis

The two targeted MAPK genes that we analyzed were obtained as pdb files from the AlphaFold Protein Structure Database (https://alphafold.ebi.ac.uk/), and their structure models were visualized using PyMOL (3.1.6.1).

## 3 RESULTS

### 3.1 Genome‐wide identification of MAPK genes in peanut

A combination of BLASTp and HMM‐based methods was used to identify MAPK family genes in three peanut species. Based on the intersection of results obtained from both BLASTP and HMM‐based methods, we identified 30 MAPK genes in *A. hypogaea* renamed *Ah_At_MAPK1*–*Ah_At_MAPK14* and *Ah_Bt_MAPK1*–*Ah_Bt_MAPK16* based on the locations of subgenomes, 16 MAPK genes in *A. duranensis* renamed *Du_MAPK1*–*Du_MAPK16*, and 15 MAPK genes in *A. ipaensis* renamed *Ip_MAPK1*–*Ip_MAPK15*.

In addition, we performed molecular characterization, including analyses of protein length, molecular weight (MW), isoelectric point, and subcellular localization, to infer the function of identified MAPK genes (Table 1). These putative *Ah_MAPK* genes were predicted to encode 368–612 amino acids in length, with putative MW ranging from 42269.8 to 69510.1 kDa, and protein isoelectric points (PI) ranging from 4.73 to 9.59. The putative *Du_MAPK* genes were predicted to encode 320–669 amino acids in length, with putative MW ranging from 37009.9 to 76405.3 kDa, and PI ranging from 4.69 to 9.47. The putative *Ip_MAPK* genes were predicted to encode 332–667 amino acids in length, with putative MW ranging from 38276.3 to 76019.9 kDa, and PI ranging from 4.58 to 9.5 (Table 1).

**TABLE 1:** The characteristics of putative mitogen‐activated protein kinase (MAPK) genes in *Arachis hypogaea*, *Arachis duranensis*, and *Arachis ipaensis*.

Name	Gene ID	Length of protein in AA (amino acid)	MW (kDa) (molecular weights)	PI (protein isoelectric points)	Subcellular localization (wolfpsort)
***A. hypogaea***					
Ah_At_MAPK1	arahy.Tifrunner.gnm2.ann2.Ah01g079400.1	612	69510.1	9.53	cyto
Ah_At_MAPK2	arahy.Tifrunner.gnm2.ann2.Ah01g371600.1	561	63724.4	9.13	cyto
Ah_At_MAPK3	arahy.Tifrunner.gnm2.ann2.Ah02g024000.1	371	42484.1	6.14	cyto
Ah_At_MAPK4	arahy.Tifrunner.gnm2.ann2.Ah03g025500.1	371	42565.6	5.85	cysk
Ah_At_MAPK5	arahy.Tifrunner.gnm2.ann2.Ah03g026600.1	369	42570.2	4.73	cyto
Ah_At_MAPK6	arahy.Tifrunner.gnm2.ann2.Ah03g476300.1	470	53683	6.66	cyto
Ah_At_MAPK7	arahy.Tifrunner.gnm2.ann2.Ah03g541700.1	606	68875.3	7.48	cyto
Ah_At_MAPK8	arahy.Tifrunner.gnm2.ann2.Ah04g035800.1	563	63962.8	8.76	cyto
Ah_At_MAPK9	arahy.Tifrunner.gnm2.ann2.Ah04g362600.1	376	43326	6.29	cyto
Ah_At_MAPK10	arahy.Tifrunner.gnm2.ann2.Ah05g215300.1	385	44089	7	cyto
Ah_At_MAPK11	arahy.Tifrunner.gnm2.ann2.Ah05g361900.1	482	55276.4	6.96	cyto
Ah_At_MAPK12	arahy.Tifrunner.gnm2.ann2.Ah07g044300.1	602	68856.5	9.59	nucl
Ah_At_MAPK13	arahy.Tifrunner.gnm2.ann2.Ah07g309600.1	600	67996.3	9.44	nucl
Ah_At_MAPK14	arahy.Tifrunner.gnm2.ann2.Ah08g009500.1	372	42634.9	6.58	nucl
Ah_Bt_MAPK1	arahy.Tifrunner.gnm2.ann2.Ah11g020100.1	612	69496	9.52	cyto
Ah_Bt_MAPK2	arahy.Tifrunner.gnm2.ann2.Ah11g412800.1	561	63754.5	9.06	cyto
Ah_Bt_MAPK3	arahy.Tifrunner.gnm2.ann2.Ah12g020900.1	371	42555.2	6.14	cyto
Ah_Bt_MAPK4	arahy.Tifrunner.gnm2.ann2.Ah13g044600.1	371	42577.6	5.85	cysk
Ah_Bt_MAPK5	arahy.Tifrunner.gnm2.ann2.Ah13g045900.1	369	42570.2	4.73	cyto
Ah_Bt_MAPK6	arahy.Tifrunner.gnm2.ann2.Ah13g505500.1	397	45404.4	5.68	cysk
Ah_Bt_MAPK7	arahy.Tifrunner.gnm2.ann2.Ah13g571200.1	608	69101.5	7.48	cyto
Ah_Bt_MAPK8	arahy.Tifrunner.gnm2.ann2.Ah13g639100.1	368	42269.8	7.95	cyto
Ah_Bt_MAPK9	arahy.Tifrunner.gnm2.ann2.Ah14g050600.1	563	63972.8	8.76	cyto
Ah_Bt_MAPK10	arahy.Tifrunner.gnm2.ann2.Ah14g426400.1	376	43326	6.29	cyto
Ah_Bt_MAPK11	arahy.Tifrunner.gnm2.ann2.Ah15g130900.1	380	43496.3	6.75	cyto
Ah_Bt_MAPK12	arahy.Tifrunner.gnm2.ann2.Ah15g423900.1	557	63890.6	6.8	cysk
Ah_Bt_MAPK13	arahy.Tifrunner.gnm2.ann2.Ah17g049300.1	606	69148.7	9.59	cyto
Ah_Bt_MAPK14	arahy.Tifrunner.gnm2.ann2.Ah17g371700.1	372	42593.8	6.58	cyto
Ah_Bt_MAPK15	arahy.Tifrunner.gnm2.ann2.Ah18g229600.1	600	68040.4	9.5	nucl
Ah_Bt_MAPK16	arahy.Tifrunner.gnm2.ann2.Ah20g525300.1	368	42269.8	7.95	cyto
***A. duranensis***					
Du_MAPK8	aradu.V14167.gnm1.ann1.Aradu.CGX1Z.1	371	42564.2	6.24	cyto
Du_MAPK12	aradu.V14167.gnm1.ann1.Aradu.J97W2.1	371	42742.5	6.61	chlo
Du_MAPK5	aradu.V14167.gnm1.ann1.Aradu.A68MB.1	607	68973.4	7.48	cyto
Du_MAPK6	aradu.V14167.gnm1.ann1.Aradu.A7MKH.1	384	43742.5	6.84	mito
Du_MAPK14	aradu.V14167.gnm1.ann1.Aradu.Q0IL1.1	368	42286.8	7.95	cyto
Du_MAPK3	aradu.V14167.gnm1.ann1.Aradu.49RUG.1	401	46137.8	6.87	cyto
Du_MAPK13	aradu.V14167.gnm1.ann1.Aradu.NJQ5G.1	383	43998.7	5.63	cyto
Du_MAPK10	aradu.V14167.gnm1.ann1.Aradu.H6YZR.1	579	66807.9	6.88	cysk
Du_MAPK2	aradu.V14167.gnm1.ann1.Aradu.44GS0.1	394	45164.3	6.41	nucl
Du_MAPK15	aradu.V14167.gnm1.ann1.Aradu.RVT4Y.1	320	37009.9	4.69	cyto
Du_MAPK1	aradu.V14167.gnm1.ann1.Aradu.271A7.1	600	68026.4	9.47	cyto
Du_MAPK7	aradu.V14167.gnm1.ann1.Aradu.B41M6.1	578	65666.8	9.13	chlo
Du_MAPK9	aradu.V14167.gnm1.ann1.Aradu.E1ZU5.1	669	76405.3	9.15	cyto
Du_MAPK16	aradu.V14167.gnm1.ann1.Aradu.WE8JU.1	566	64303.2	8.86	cyto
Du_MAPK4	aradu.V14167.gnm1.ann1.Aradu.93Y1F.1	600	67996.3	9.44	nucl
Du_MAPK11	aradu.V14167.gnm1.ann1.Aradu.IP5K7.1	371	42565.6	5.85	cysk
***A. ipaensis***					
Ip_MAPK7	araip.K30076.gnm1.ann1.Araip.CL071.1	372	42603.9	6.58	cyto
Ip_MAPK15	araip.K30076.gnm1.ann1.Araip.Y7IQI.1	561	63754.5	9.06	cyto
Ip_MAPK10	araip.K30076.gnm1.ann1.Araip.P1N43.1	667	76019.9	9.47	cyto
Ip_MAPK2	araip.K30076.gnm1.ann1.Araip.6I993.1	380	43496.3	6.75	cyto
Ip_MAPK4	araip.K30076.gnm1.ann1.Araip.96FUL.1	563	63972.8	8.76	cyto
Ip_MAPK6	araip.K30076.gnm1.ann1.Araip.AT3RC.1	368	42269.8	7.95	cyto
Ip_MAPK5	araip.K30076.gnm1.ann1.Araip.AH33F.1	448	51476.6	6.51	plas
Ip_MAPK8	araip.K30076.gnm1.ann1.Araip.IL3I2.1	371	42577.6	5.85	cysk
Ip_MAPK13	araip.K30076.gnm1.ann1.Araip.U4Z8Q.1	600	68040.4	9.5	nucl
Ip_MAPK14	araip.K30076.gnm1.ann1.Araip.WRI31.1	387	44044.8	6.79	mito
Ip_MAPK3	araip.K30076.gnm1.ann1.Araip.8K6RH.1	332	38276.3	4.58	cyto
Ip_MAPK1	araip.K30076.gnm1.ann1.Araip.32FLV.1	371	42555.2	6.14	cyto
Ip_MAPK9	araip.K30076.gnm1.ann1.Araip.LMV71.1	615	69814.4	7.71	cyto
Ip_MAPK11	araip.K30076.gnm1.ann1.Araip.RV49X.1	655	74155.5	9.39	E.R.
Ip_MAPK12	araip.K30076.gnm1.ann1.Araip.TJ3I8.1	557	63880.6	6.8	cysk

Subcellular localization prediction has revealed that within *A. hypogaea*, FOUR putative *Ah_MAPKs* were located in the cytoskeleton, 22 in the cytoplasm, and four in the nucleus. In the case of *A. duranensis*, two *Du_MAPKs* were identified in the chloroplast, two in the cytoskeleton, nine in the cytoplasm, one in the mitochondrion, and two in the nucleus. As for *A. ipaensis*, two *Ip_MAPKs* were located in the cytoskeleton, nine in the cytoplasm, one in the endoplasmic reticulum, one in the mitochondrion, one in the nucleus, and one in the plastids (Table 1).

### 3.2 Phylogenetic analysis of MAPK genes

**FIGURE 1:**
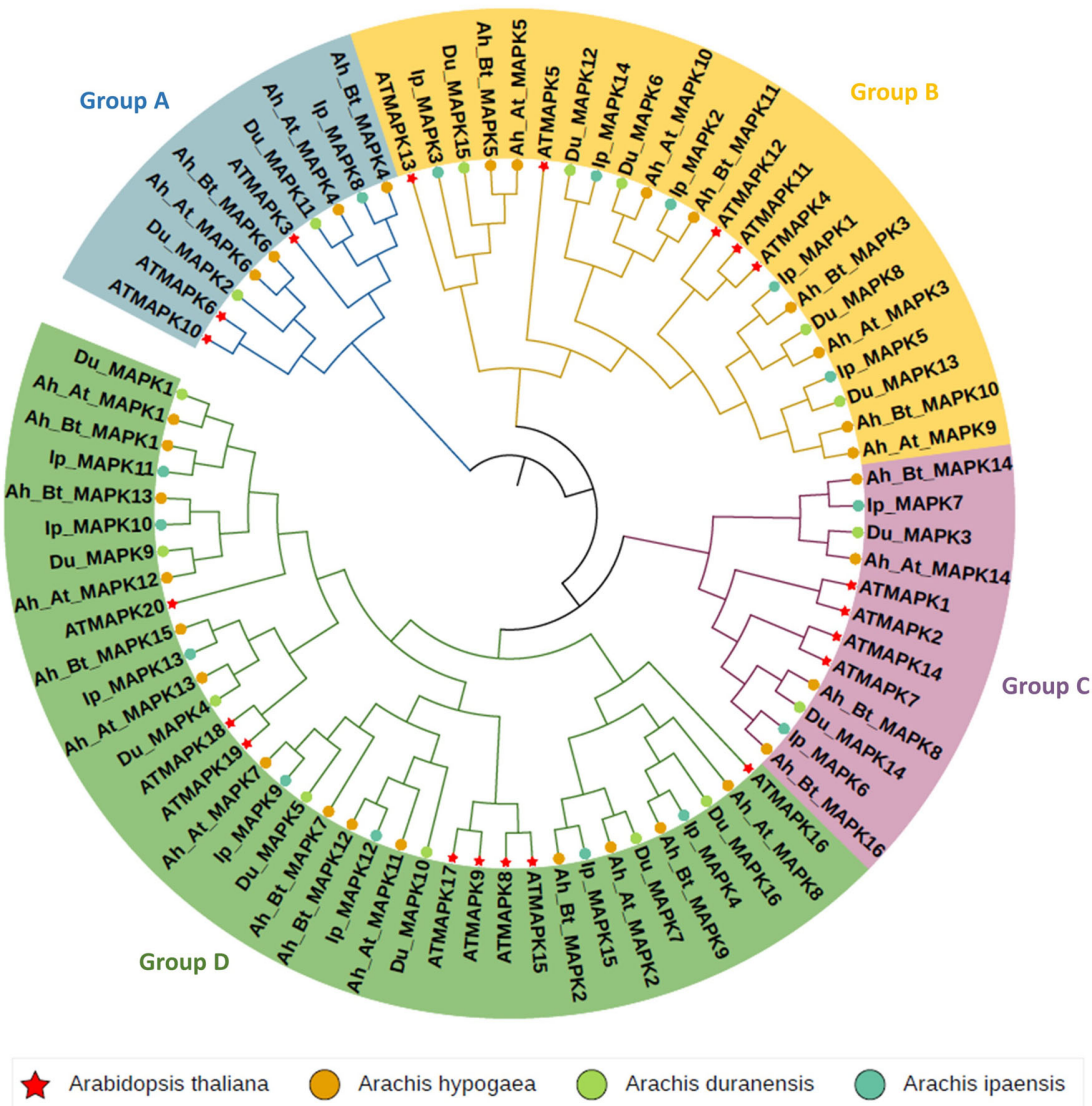
Phylogenetic relationship of putative mitogen‐activated protein kinase (MAPK) genes in *Arachis ipaensis*, *Arachis duranensis*, *Arachis hypogaea*, and *Arabidopsis thaliana*. Different color blocks within the tree denote distinct groups, five‐points stars represent. Different shapes preceding the MAPK gene represent different species.

To better investigate the evolutionary relationship among the MAPK proteins, a maximum likelihood phylogenetic tree was constructed to determine the evolutionary relationship of MAPK genes by using amino acid sequences of 16, 15, and 30 identified MAPK genes in *A. duranensis*, *A. ipaensis*, and *A. hypogaea*, respectively, and with corresponding 20 *AtMAPKs* of *A. thaliana* (Yao et al., 2022). In plants, MAPK proteins have diverged into four major subfamilies (A–D), and sequence comparison containing the amino acid motif TEY can be classified into three groups, A, B and C, whereas sequence comparison containing the amino acid motif TDY forms a more distant group D (Ichimura et al., 2002). This categorization is consistent with previous studies conducted on *Arabidopsis*, maize, and *Fagopyrum tataricum* (Ichimura et al., 2002; Y. K. Liu et al., 2013; Yao et al., 2022). Our phylogenetic analysis also showed the peanut MAPK genes were divided into four distinct groups (groups A, B, C, and D) specifically together with their MAPK orthologs in *Arabidopsis* (Figure 1). Group A contained four *Ah_MAPKs*, one *Ip_MAPK*, and two *Du_MAPKs*, clustering with the well‐characterized *AtMAPK3/6/10*. Group B comprised eight *Ah_MAPKs*, five *Ip_MAPKs*, and five *Du_MAPKs*, grouping with *AtMAPK4/5/11/12/13*. Group C included four *Ah_MAPKs*, two *Ip_MAPKs*, and two *Du_MAPKs*, associated with *AtMAPK1/2/7/14*. Group D was the largest subfamily, containing 14 *Ah_MAPKs*, seven *Ip_MAPKs*, and seven *Du_MAPKs*, clustering with *AtMAPK8/9/15/16/18/19/20*. The distribution pattern showed group D contained the most members, followed by groups B and C, while group A had the fewest genes.

### 3.3 Gene structure and motif composition of putative MAPKs in peanut

**FIGURE 2:**
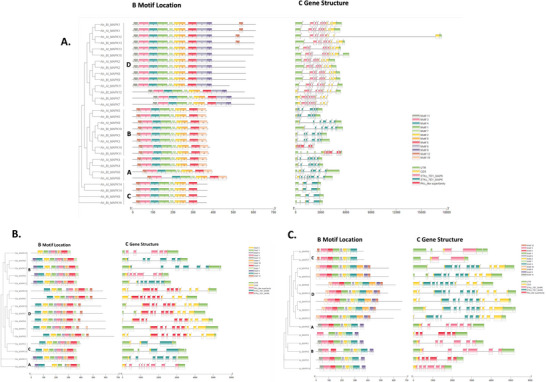
(A) Analysis of the structure and conserved motifs of mitogen‐activated protein kinase (MAPK) genes in *Arachis hypogaea;* (B) analysis of the structure and conserved motifs of MAPK genes in *Arachis duranensis;* (C) analysis of the structure and conserved motifs of MAPK genes in *Arachis ipaensis*.

To further analyze the potential roles of putative MAPKs, we conducted exon–intron structures, motif distribution, and conserved domain analysis. The exon/intron structures, including the number, length, and arrangement of exons and introns, were analyzed to provide insights into the gene structure diversity and phylogenetic relationship among members of the gene family. Structures and splice phases (0, 1, or 2) of introns/exons were determined by the alignment of genomic DNA with full‐length cDNA of MAPKs. The exon/intron structures and motif location of putative MAPK genes can also be categorized into four groups, based on their phylogenetic relationships (Figure 2). Gene structure analysis showed that MAPK gene family structure was relatively complex and putative MAPK genes from different groups exhibit significantly different exon/intron structures, while the MAPK genes were highly conserved within the same group. The gene structures of putative MAPK genes in groups A, B, and C exhibit more conservation compared to those in group D, which display a complex arrangement of exons and introns.

The sequence characteristics of peanut MAPK genes were analyzed using MEME software, and a total of 12 distinct motifs were predicted. The motifs are more conserved for each of the three peanut species. For *A. hypogaea*, those 10 conserved motifs with the same order and orient, including motifs 11, 3, 4, 1, 7, 2, 8, 5, 6, and 9, were identified in of the all D group, and 8 conserved motifs, including motifs 12, 3, 4, 1, 7, 2, 8, and 5, were identified in all of A, B, and C groups (Figure 2A). Furthermore, motifs 3, 4, 1, 7, 2, 8, and 5 were identified in all MAPK genes of *A. hypogaea*. For *A. duranensis*, the combination of conserved motifs (motifs 4, 2, 1, 6, 3, 11, 8, 10, 5, and 7) was identified in nearly all MAPK genes (Figure 2B), except *Du_MAPK15* (missing motif 4), *Du_MAPK10* (missing motif 11), and *Du_MAPK2* (missing motifs 6 and 3). For *A. ipaensis*, the conserved combination of motifs (motifs 3, 5, 1, 6, 2, 7, and 4) was identified in nearly all MAPK genes except *Ip_MAPK3* (missing motif 5) (Figure 2C). Conserved domain analysis revealed that groups A, B, and C contain STKc_TEY_MAPK domain, while group D contains STKc_TDY_MAPK domain. The differences in motif composition between group D and groups A, B, and C could have significant effects on their biological functions. These potential effects need to be investigated further through biological experiments.

### 3.4 Chromosomal distribution and collinearity analysis of putative MAPKs

**FIGURE 3:**
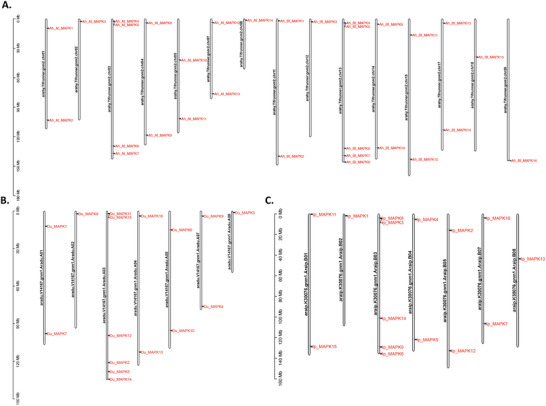
(A) Chromosomal distribution of identified mitogen‐activated protein kinase (MAPK) genes in *Arachis hypogaea;* (B) chromosomal distribution of identified MAPK genes in *Arachis duranensis;* (C) chromosomal distribution of identified MAPK genes in *Arachis ipaensis*.

**FIGURE 4:**
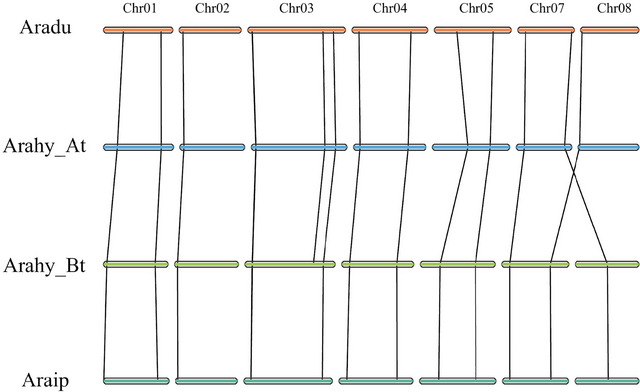
Collinearity analysis of identified mitogen‐activated protein kinase (MAPK) genes between *Arachis duranensis*, *Arachis ipaensis*, and A, B subgenomes in *Arachis hypogaea*.

The majority of MAPK genes were located on the proximate or the distal ends of the chromosomes among three peanut species. In *A. hypogaea*, 14 MAPK genes were present on all chromosomes except chromosomes 6, 9, and 10 for A subgenome and 16 MAPK genes were present on all chromosomes except chromosomes 16 and 19 for B subgenome. For the two diploid peanut species, the 15 and 16 MAPK genes were present on all chromosomes except chromosomes 6, 9, and 10 for both *A. duranensis* and *A. ipaensis*. Chromosome 3 holds the most MAPK genes in both diploid species, and this finding is also consistent with the At and Bt subgenomes of *A. hypogaea* (Figure 3). Collinearity analysis of the MAPK gene family among *A. ipaensis*, *A. duranensis*, and the subgenomes of *A. hypogaea* (At and Bt) shows that most of MAPK genes are largely conserved across these genomes. However, two notable homologous gene pairs located on chromosomes 7 and 8 exhibited divergence between the diploid species *A. duranensis* and *A. ipaensis* (Figure 4). This divergence was retained in the At and Bt subgenomes of *A. hypogaea* after hybridization and polyploidization, indicating that structural variations or evolutionary events in these gene pairs occurred prior to the formation of the tetraploid genome.

### 3.5 Estimation of nonsynonymous (Ka) and synonymous (Ks) substitutions per site and their ratios

**FIGURE 5:**
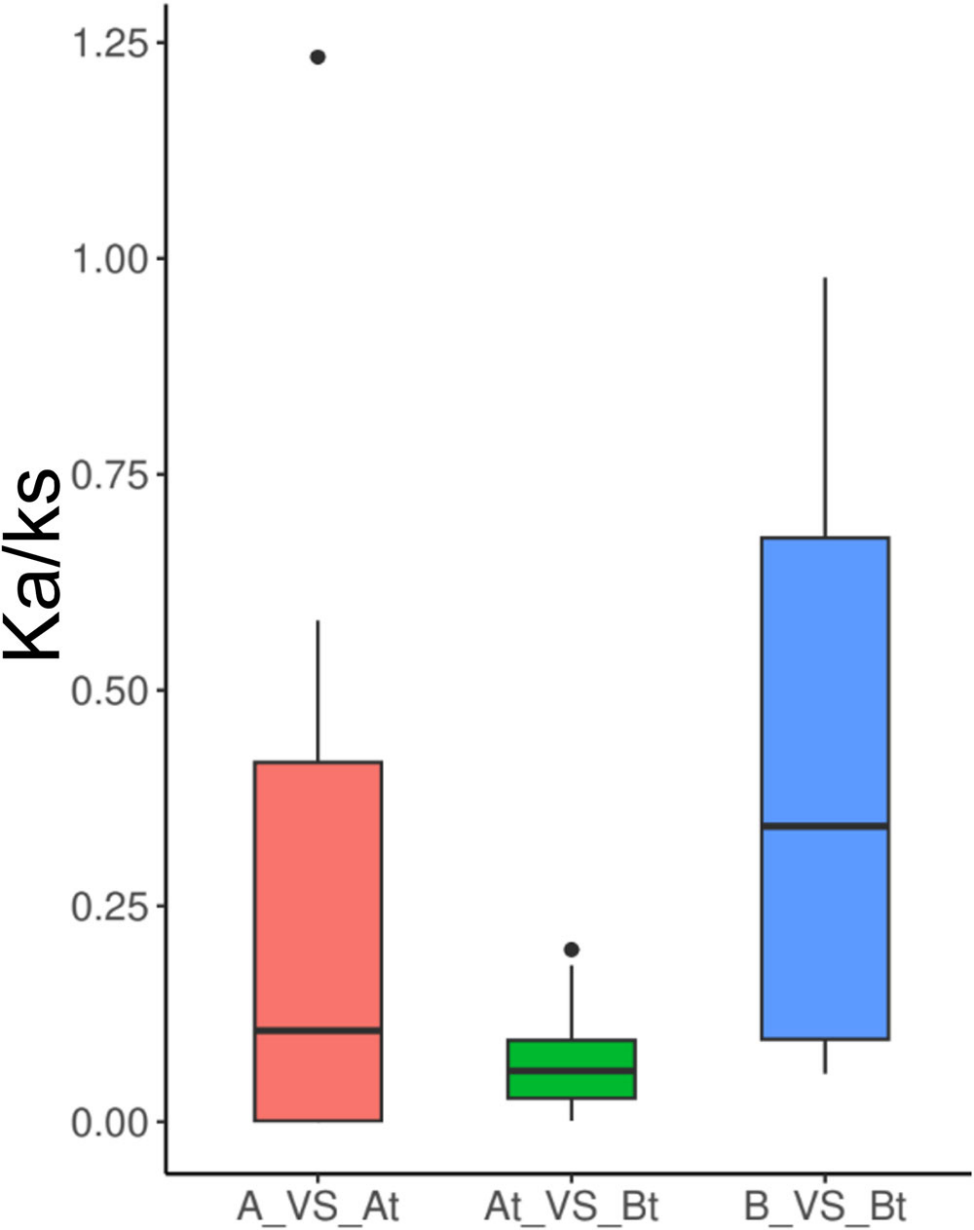
Selection pressure analysis. Ka/Ks ratio comparisons represented by box plots in different colors: the red box represents the comparison between the A subgenome of *Arachis hypogaea* and *Arachis duranensis*, the green box represents the comparison within the subgenomes (At vs. Bt) of *A. hypogaea*, and the blue box represents the comparison between the B subgenome of *A. hypogaea* and *Arachis ipaensis*.

To analyze the selection pressures of MAPK genes on two sub‐genomes of allotetraploid peanut *A. hypogaea* and their corresponding two diploid peanut species *A. duranensis and A. ipaens*is, Ka/Ks per site and their ratios were calculated. As illustrated in Figure 5, the comparisons include the A subgenome of *A. hypogaea* versus *A. duranensis* (A vs. At), the B subgenome of *A. hypogaea* and *A. ipaensis* (B vs. Bt), and the internal comparison within *A. hypogaea* between the At and Bt subgenomes (At vs. Bt). The majority of the Ka/Ks ratio comparisons exhibited values below 1 indicating that purifying selection of MAPK genes occurred in both diploid and polyploid peanut species. This pattern suggests a strong conservation of essential genetic functions, critical for maintaining stability and functionality within the plant. However, an exception was observed in the pair *Du_MAPK13* and *Ah_At_MAPK9*, which showed a ratio above 1, as detailed in Table S1. This indicates possible positive selection, highlighting a potential adaptive genetic evolution in this gene pair. Additionally, we observed that compared to the A versus At, the MAPK genes in the B versus Bt are more conserved, with fewer mutations occurring.

### 3.6 Cis‐acting element analysis of putative MAPKs

**FIGURE 6:**
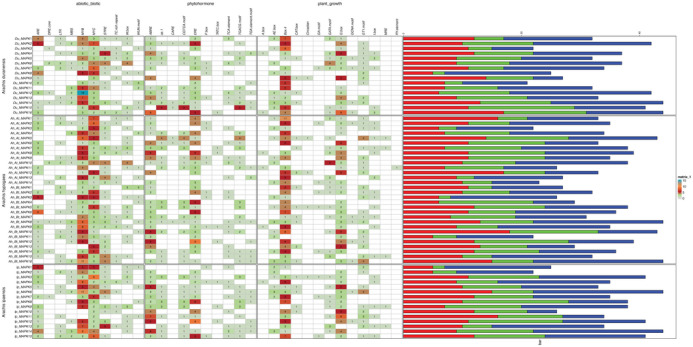
Cis‐acting element prediction analysis in identified mitogen‐activated protein kinase (MAPK) genes. The heatmap represents the presence (colored boxes) or absence (white boxes) of various cis‐acting elements in the promoter regions of MAPK genes. The stacked bar chart on the right depicts the distribution of cis‐acting elements classified into three categories: stress‐responsive (abiotic/biotic) in blue, plant growth and developmental in red, and phytohormone responsive in green.

Cis‐acting element analysis was conducted to further explore the potential function of transcriptional regulation of the identified MAPKs in peanuts. We extracted 2000 bp upstream sequences of the start codon of identified MAPKs to analyze cis‐acting elements in the promoter regions. Cis‐element analysis revealed the presence of various potential regulatory elements in the promoter regions of MAPK genes from *A. hypogaea*, its potential progenitor *A. duranensis*, and the other wild relative *A. ipaensis*. A total of 1002, 545, and 531 cis‐acting elements were identified in *A. hypogaea*, *A. duranensis*, and *A. ipaensis*, respectively. These cis‐acting elements could be classified into three major types, stress responsive (abiotic/biotic), phytohormone responsive, and plant growth and developmental (Table S2; Figure 6). In the first category related to stress responses, 415, 235, and 228 relevant elements were identified in *A. hypogaea*, *A. duranensis*, and *A. ipaensis*, respectively. Notably, the MYB and MYC elements have been previously reported to be involved in drought stress responses (Abe et al., 1997), and the MYB and MYC accounted for more than 55% of stress‐responsive elements across all three species. In the second category of phytohormone‐responsive elements, a total of 269, 147, and 149 cis‐acting elements were identified in *A. hypogaea*, *A. duranensis*, and *A. ipaensis*, respectively. Within this category, two important stress‐responsive cis‐regulatory elements ABRE (abscisic acid‐responsive element) and ERE (ethylene‐responsive element) were identified and they accounted for more than 54% of phytohormone‐responsive elements across all three species. The third category comprised elements associated with plant growth and development, with 318, 163, and 154 elements detected in *A. hypogaea*, *A. duranensis*, and *A. ipaensis*. Among these, two crucial light‐responsive elements Box‐4 and G‐Box were found, which accounted for 61% of plant growth and development elements across all species. The occurrence frequencies of these key cis‐regulatory elements, such as MYB, ABRE, and Box‐4, were highly consistent among the three peanut species. This suggests a high degree of conservation of MAPK genes in peanut species and their close association with stress responses. These findings lay the foundation for further elucidating the upstream regulatory mechanisms governing MAPK gene expression in peanut.

### 3.7 Expression analysis of MAPK genes response to drought stress in *A. hypogaea*

To investigate the expression patterns of identified MAPK genes under drought stress conditions, we conducted RNA‐seq analysis of four peanut genotypes (two drought‐tolerant: C76‐16 and 587; two drought‐susceptible: Tifrunner and 506) under control and drought treatment conditions. PCA of RNA‐seq data revealed that drought treatment and genotype‐specific responses are the main drivers of transcriptional variation among peanut samples (Figure S1). The first principal component (5.6), explaining 29.63% of variance, distinguished drought‐treated samples from controls. The second principal component (PC2), accounting for 11.06% of variance, reflected genotypic differences that were distinct under drought conditions, with drought‐tolerant genotypes (C76‐16 and 587) separating from susceptible genotypes (506 and Tifrunner), whereas there was no significant genotypic separation under control conditions.

**FIGURE 7:**
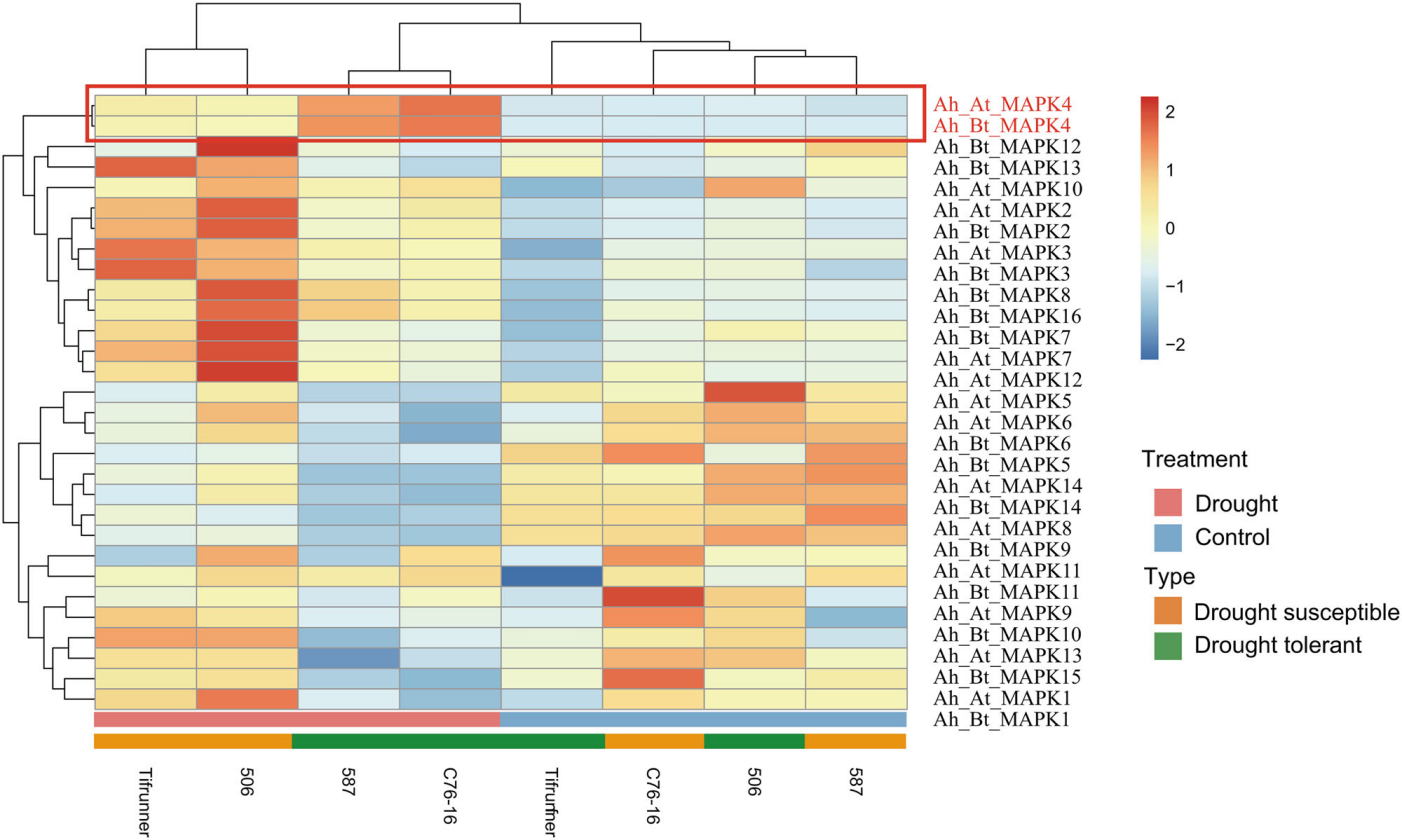
Heatmap of mitogen‐activated protein kinase (MAPK) gene expression patterns in drought‐tolerant and drought‐susceptible peanut genotypes under control and drought conditions.

Hierarchical clustering of MAPK gene expression (Figure 7) revealed distinct expression patterns in response to drought stress. Notably, *Ah_At_MAPK4* and *Ah_Bt_MAPK4* showed remarkable upregulation under drought conditions, particularly in drought‐tolerant genotypes. These genes displayed high expression levels under drought conditions compared to low expression under control conditions, suggesting their potential role in drought response mechanisms.

**FIGURE 8:**
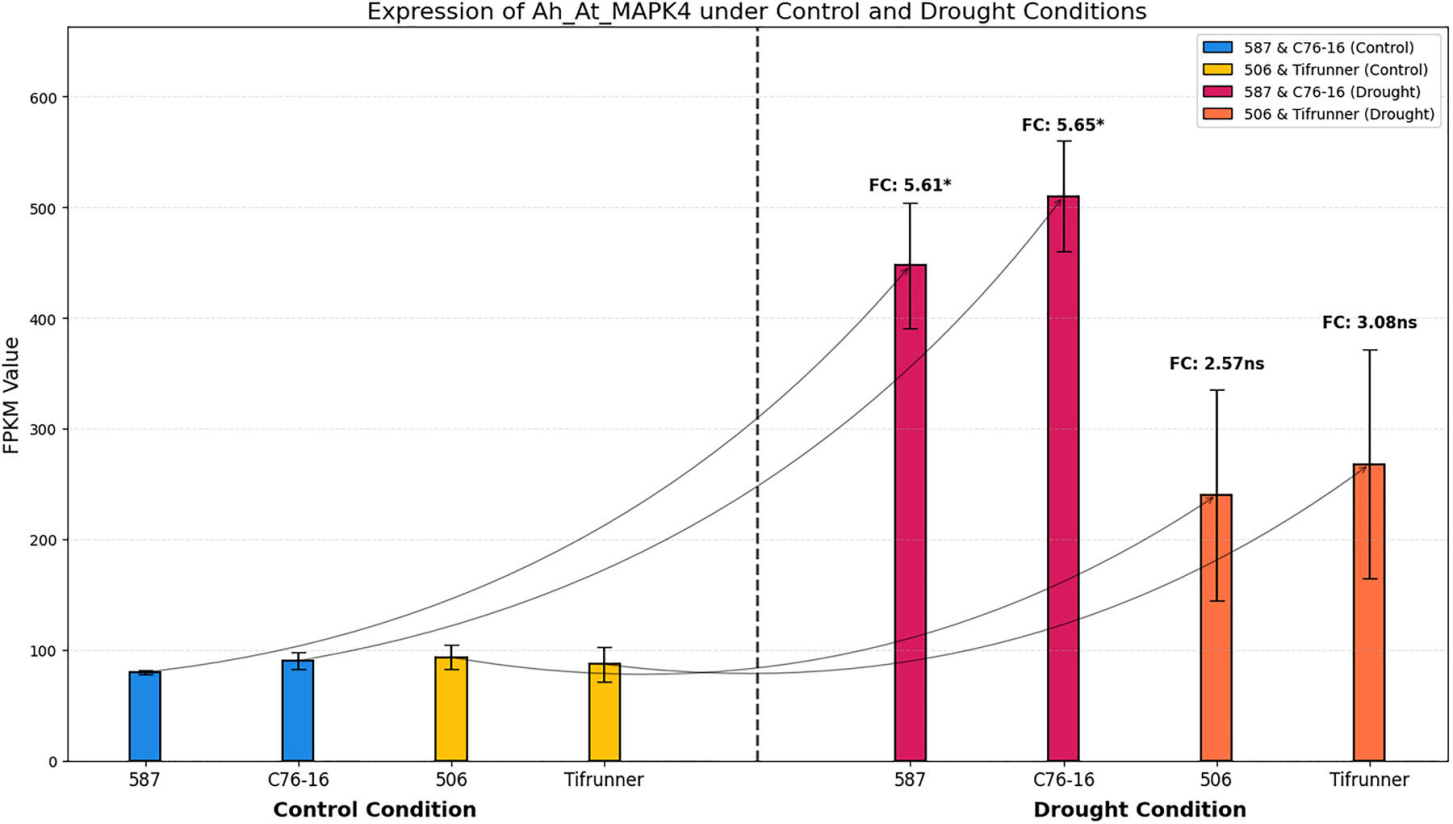
Expression analysis of *Ah_At_MAPK4* under control and drought conditions. Error bars indicate standard error of the mean (SEM). Fold change (FC) values with significance levels are shown above each drought treatment bar (*p < 0.05, ns = not significant). FPKM, fragments per kilobase of transcript per million mapped reads.

**FIGURE 9:**
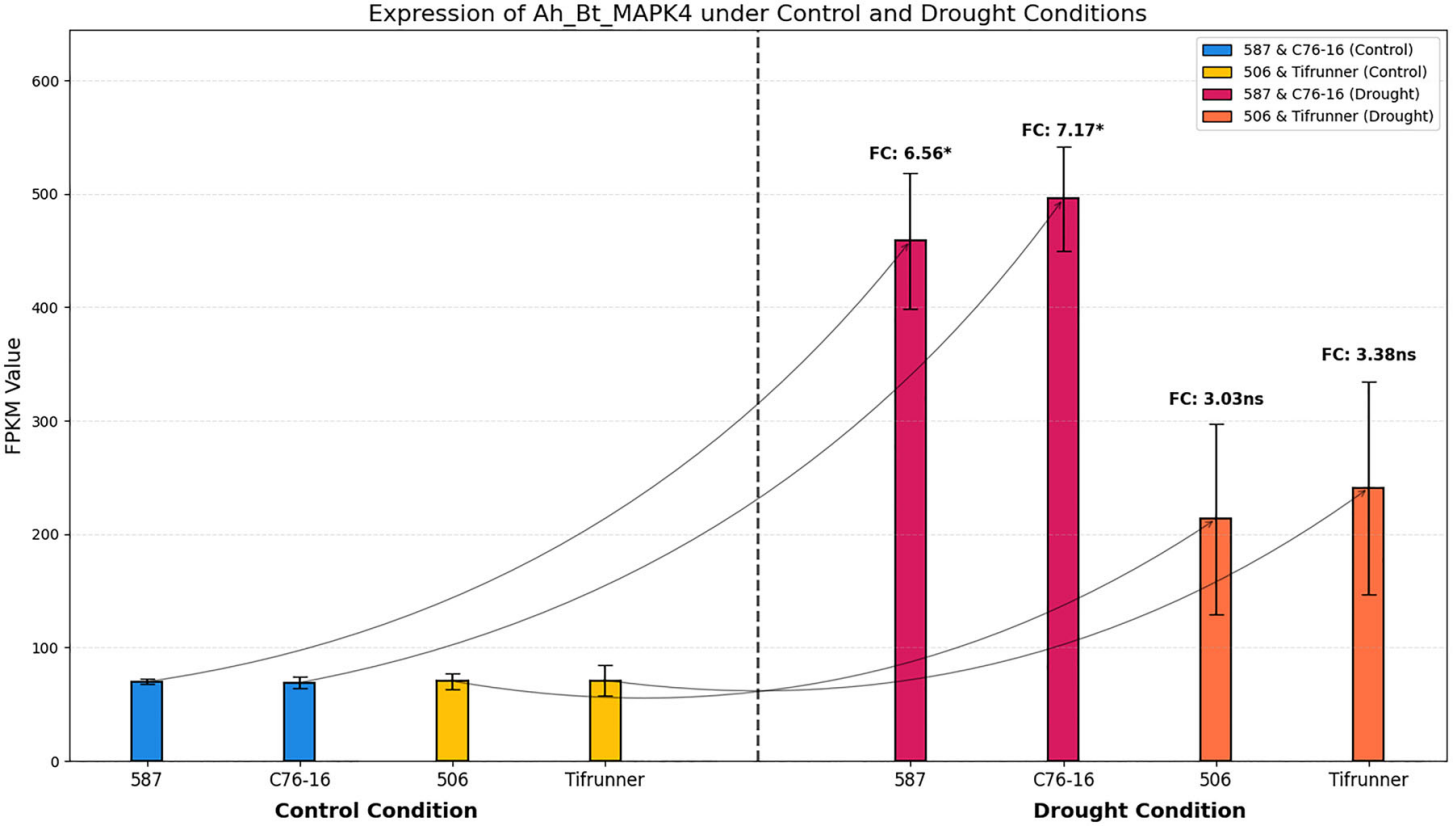
Expression analysis of *Ah_Bt_MAPK4* under control and drought conditions. Error bars indicate standard error of the mean (SEM). Fold change (FC) values with significance levels are shown above each drought treatment bar (*p < 0.05, **p < 0.01, ***p < 0.001, ns = not significant). FPKM, fragments per kilobase of transcript per million mapped reads.

To quantitatively assess the expression patterns observed in the hierarchical clustering heatmap, we examined the FPKM values of *Ah_At_MAPK4* and *Ah_Bt_MAPK4* across individual biological replicates (Figures 8 and 9). This quantitative analysis allowed us to determine fold‐change values and statistical significance of differential expression between drought‐treated and control samples within each genotype, as well as to compare induction levels between drought‐tolerant and drought‐susceptible genotypes. The results demonstrated that under control conditions, expression levels of these two genes were similar between drought‐tolerant and drought‐susceptible genotypes. *Ah_At_MAPK4* showed baseline expression values of 79.82 ± 1.88, 90.20 ± 7.21, 93.21 ± 10.99, and 86.90 ± 15.37 FPKM in 587, C76‐16, 506, and Tifrunner, respectively, under control conditions. Similarly, *Ah_Bt_MAPK4* exhibited comparable expression levels across all genotypes under control conditions: 69.92 ± 2.37, 69.16 ± 4.87, 70.42 ± 7.21, and 71.17 ± 13.07 FPKM, respectively. However, under drought stress, both genes showed significant upregulation in all genotypes, with substantially higher induction in drought‐tolerant genotypes compared to drought‐susceptible ones. *Ah_At_MAPK4* expression increased to 447.74 ± 56.64 and 510.05 ± 49.89 FPKM in drought‐tolerant genotypes 587 and C76‐16, representing 5.61‐fold (*p* = 0.0228) and 5.65‐fold (*p* = 0.0125) increases, respectively. In contrast, in drought‐susceptible genotypes 506 and Tifrunner, expression levels reached only 239.86 ± 95.62 and 267.75 ± 103.33 FPKM, corresponding to 2.57‐fold (*p* = 0.2640) and 3.08‐fold (*p* = 0.2202) increases (not statistically significant). Similarly, *Ah_Bt_MAPK4* showed significant upregulation of 6.56‐fold (*p* = 0.0226) and 7.17‐fold (*p* = 0.0108) in drought‐tolerant genotypes 587 and C76‐16, with FPKM values of 458.61 ± 59.67 and 495.60 ± 46.15, respectively. In drought‐susceptible genotypes 506 and Tifrunner, with FPKM values of 213.30 ± 83.90 and 240.66 ± 93.90, the expression increases were more moderate at 3.03‐fold (*p* = 0.2300) and 3.38‐fold (*p* = 0.2110).

Combining the results from the heatmap (Figure 7) and FPKM analysis (Figures 8 and 9), we observed that *Ah_At_MAPK4* and *Ah_Bt_MAPK4* exhibit significant drought‐induced expression patterns. These homologous genes showed similar expression levels in both drought‐tolerant and drought‐susceptible genotypes under normal conditions. However, under drought stress, they were upregulated in all genotypes, with significantly higher induction in drought‐tolerant genotypes compared to drought‐susceptible ones. These findings indicate that these MAPK genes not only respond to drought stress but also exhibit enhanced responsiveness in drought‐tolerant materials, suggesting their important role in peanut drought tolerance mechanisms.

### 3.8 WGCNA reveals drought‐responsive gene modules

**FIGURE 10:**
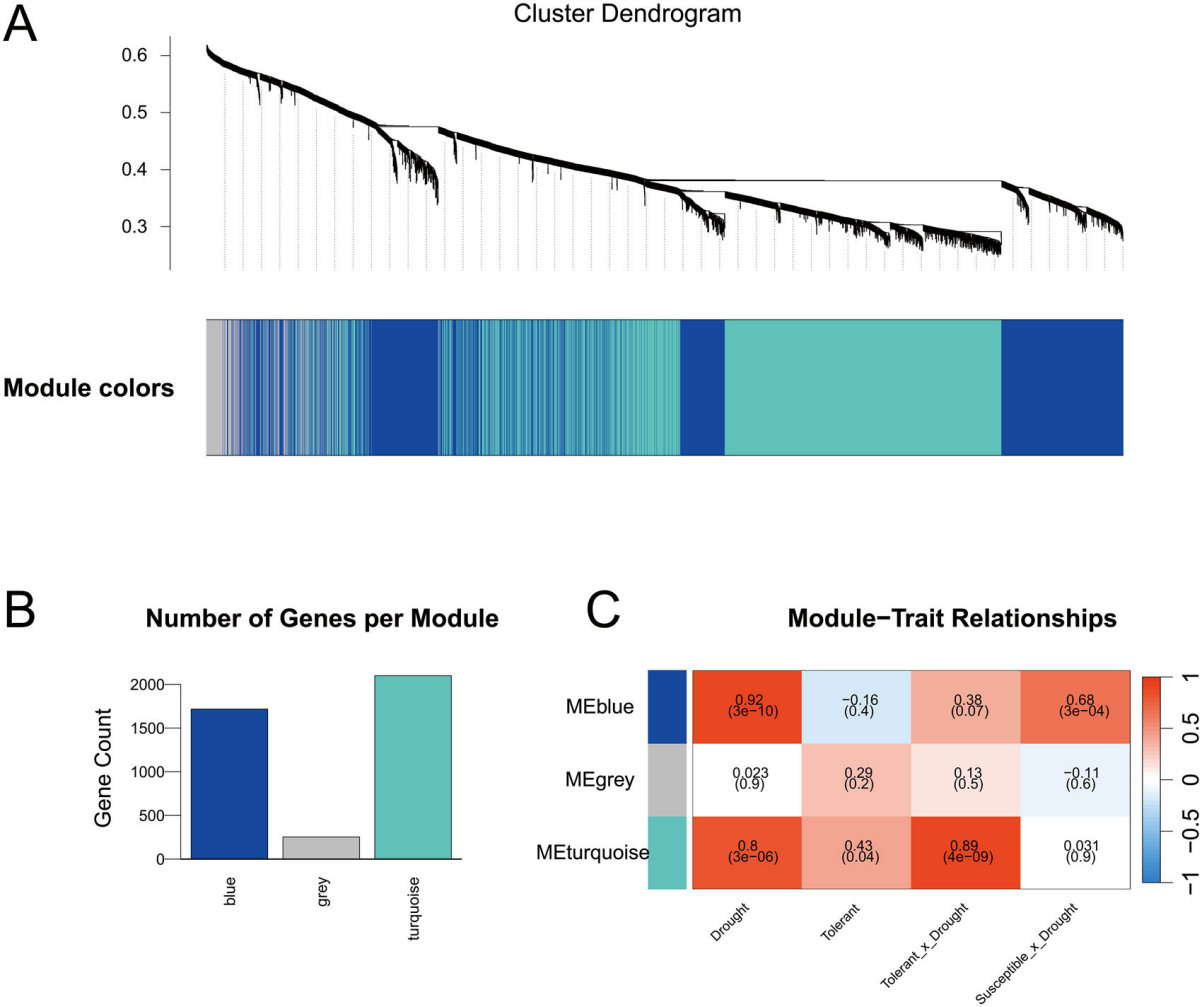
Weighted gene co‐expression network analysis in drought‐stress response of peanut leaves. (A) Hierarchical clustering dendrogram and module colors of differentially expressed genes (DEGs) and mitogen‐activated protein kinases (MAPKs). (B) Distribution of gene counts in modules and (C) module‐trait relationship heatmap of three modules.

To investigate the regulatory networks associated with *Ah_At_MAPK4* and *Ah_Bt_MAPK4*, WGCNA was conducted. Hierarchical clustering of normalized expression profiles identified gene modules, grouping genes with similar co‐expression patterns. Dynamic tree cutting with a minimum module size of 30 was performed, followed by merging of modules with highly similar eigengenes at a cut height of 0.25, generating three distinct modules colored blue, gray, and turquoise (Figure 10A), with the turquoise module encompassing 2099 genes, followed by blue with 1717 genes, and gray containing 253 genes (Figure 10B; Tables S3–S5). Soft‐thresholding power of 12 was selected to ensure scale‐free network topology during module construction (Figure S2). Notably, both drought‐responsive MAPK genes, *Ah_At_MAPK4* and *Ah_Bt_MAPK4*, were localized within the turquoise module, suggesting this module's important role in mediating drought stress responses. Correlation analysis between module eigengenes and drought‐related phenotypic traits revealed the turquoise module as the most strongly positively correlated with the tolerant × drought interaction (*r* = 0.89, *p* < 0.001), indicating its importance in the drought tolerance response. The blue module also showed moderate correlation with drought traits, while the gray module lacked significant associations (Figure 10C).

**FIGURE 11:**
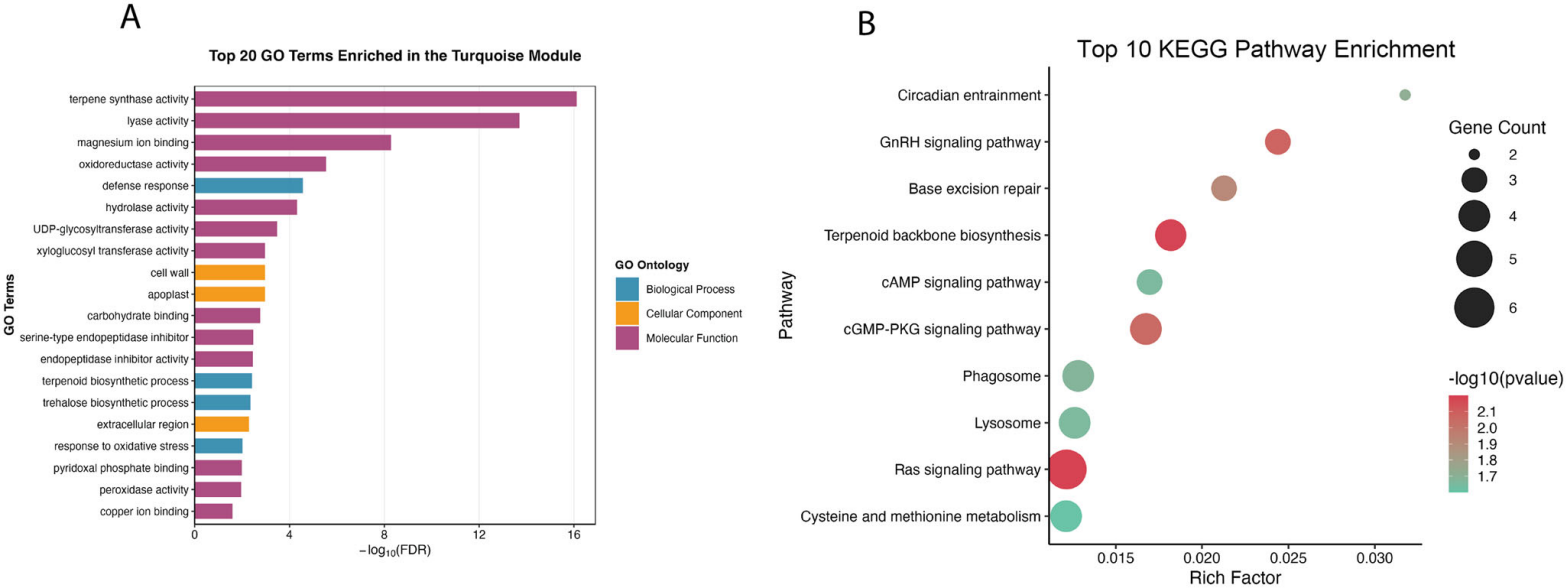
Functional enrichment analysis of genes in the turquoise module. (A) Top 20 gene ontology (GO) terms enriched in the turquoise module. (B) Top 10 kyoto encyclopedia of genes and genomes (KEGG) pathways enriched in the turquoise module.

To demonstrate the biological significance of the turquoise module, we performed comprehensive GO and KEGG analysis. GO analysis revealed significant overrepresentation of molecular functions associated with drought stress responses and secondary metabolite biosynthesis (Figure 11A; Table S6). The turquoise module was enriched in terpene synthase activity, lyase activity, and magnesium ion binding, suggesting roles in terpenoid biosynthesis and cofactor‐dependent enzymatic processes related to stress tolerance. KEGG pathway analysis identified enrichment of key metabolic and signaling pathways (Figure 11B). Terpenoid backbone biosynthesis was among the enriched pathways, aligning directly with the GO enrichment for terpene synthase activity. In addition, the simultaneous enrichment of cysteine and methionine metabolism alongside key signaling networks such as Ras, cAMP, and cGMP‐PKG pathways suggests a coordinated regulatory architecture.

### 3.9 Hub gene network analysis in the target modules

**FIGURE 12:**
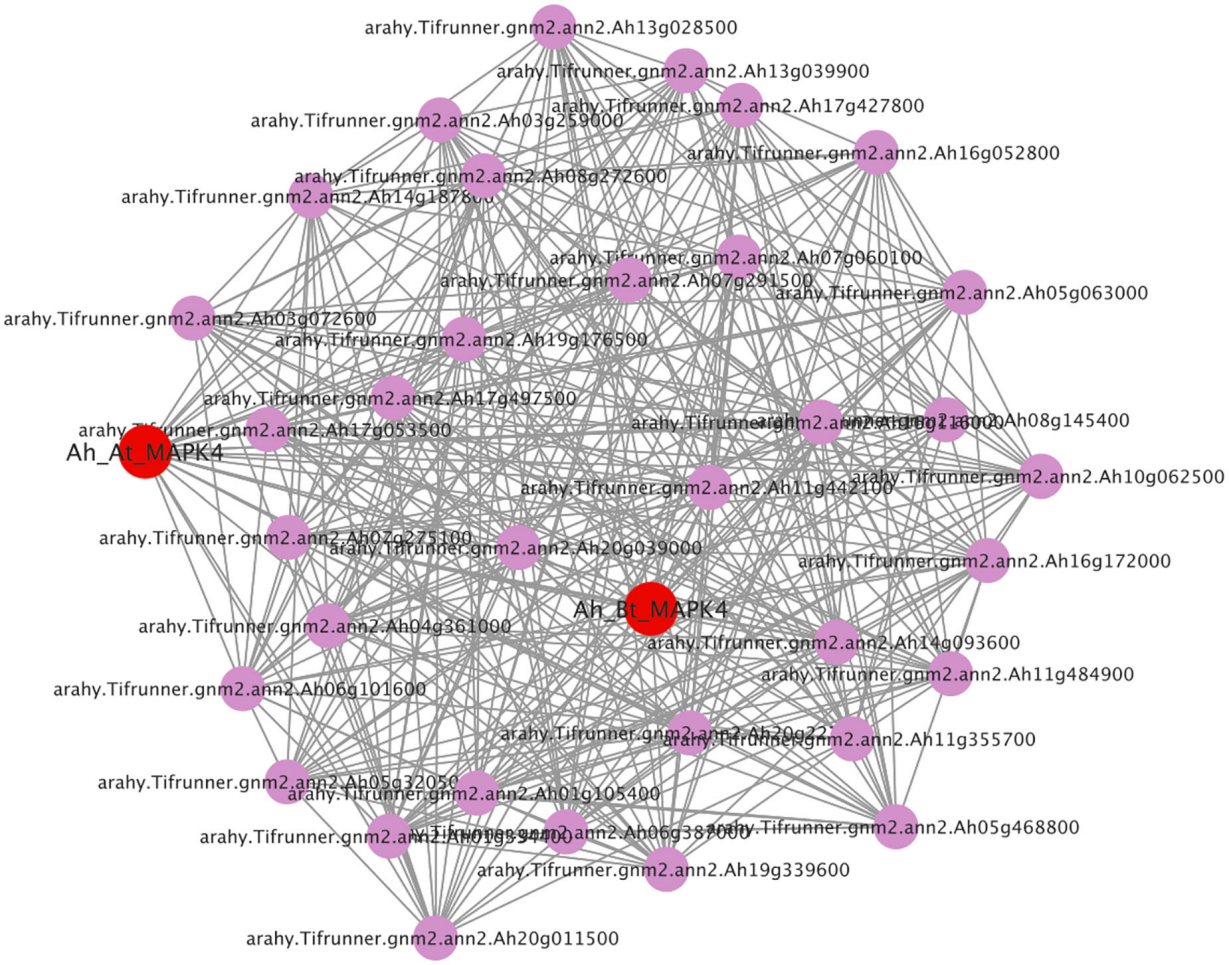
Interaction networks of hub genes in the turquoise module.

To contextualize *Ah_At_MAPK4* and *Ah_Bt_MAPK4* within the drought‐associated turquoise module, 36 hub candidates were selected based on high connectivity and used to build a co‐expression network together with these two MAPK genes (Figure 12; Table S7). GO analysis of the hubs identified calmodulin binding as significantly enriched (*p* = 6.99 × 10^−4^; false discovery rate = 0.007), implicating a calcium‐dependent signaling as a key component downstream of MAPK activation (Table S7). KEGG results revealed enrichment for diterpenoid and gibberellin biosynthetic pathways, consistent with stress‐induced secondary metabolism (Table S7). Co‐expression network analysis showed that *Ah_At_MAPK4* and *Ah_Bt_MAPK4* are strongly connected to multiple hub genes within the turquoise module. The enrichment of calmodulin binding among hub genes highlights the potential importance of calcium‐dependent signaling in this drought‐responsive network.

### 3.10 Structural modeling of MAPK proteins

**FIGURE 13:**
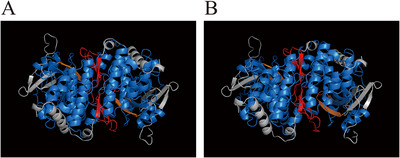
Predicted 3D structures of *Ah_At_MAPK4* (A) and *Ah_Bt_MAPK4* (B). The kinase domain is shown in marine blue, the predicted ATP‐binding site in orange, and the activation loop containing the Thr‐Glu‐Tyr (TEY) motif in red.

To further investigate the functional architecture of the key drought‐responsive genes, the 3D protein structures of *Ah_At_MAPK4* and *Ah_Bt_MAPK4* were modeled using the AlphaFold database. The predicted models for both proteins were highly reliable, with average predicted local distance difference test scores of 89.5 and 89.69, respectively. Both proteins displayed the canonical bilobed kinase fold characteristic of MAPKs (Figure 13). Key functional features were clearly resolved, including an ATP‐binding pocket in the cleft between the N‐ and C‐terminal lobes and an accessible activation loop containing the conserved TEY motif. These predicted structural features are consistent with the catalytic and regulatory architecture of MAPKs and provide a structural basis for their role as active signal‐transducing kinases in response to drought stress.

## 4 DISCUSSION

In this study, we conducted a whole genome wide identification of MAPK in three peanut species, two wild diploid species, *A. duranensis*, *A. ipaensis*, and one cultivated tetraploid species, *A. hypogaea*. A total of 61 MAPK genes were identified in the three peanut genomes, with 30 in *A. hypogaea*, 16 in *A. duranensis*, and 15 in *A. ipaensis*.

Characterization analysis of the identified MAPK genes revealed differences in parameters such as protein length, MW, and protein isoelectric point among the three species, reflecting the diversification of MAPKs during evolution. Subcellular localization prediction showed that peanut MAPKs are primarily localized in the cytoplasm, specifically 22 *Ah_MAPKs*, nine *Du_MAPKs*, and nine *Ip_MAPKs*. This is consistent with their roles in intracellular signal transduction.

We conducted phylogenetic analysis to understand the evolutionary relationship among the identified MAPK genes in peanut species and *A. thaliana*. In plants, MAPK genes have diverged into four subfamilies based on the conserved TEY/TDY motifs in the activation loop region (Ichimura et al., 2002; Y. K. Liu et al., 2013; Yao et al., 2022). In this study, we classified the 30 *Ah_MAPKs*, 16 *Du_MAPKs*, and 15 *Ip_MAPKs* into four major subfamilies (A, B, C, and D), consistent with the classification of MAPKs in previous reports. In group A, four *AhMAPKs*, one *IpMAPK*, and two *DuMAPKs* clustered together with the well‐studied *Arabidopsis AtMAPK3*, *AtMAPK6*, and *AtMAPK10*. *AtMAPK3* and *AtMAPK6* have been shown to be involved in plant responses to biotic stress, such as against *Botrytis cinerea* (Han et al., 2010), and abiotic stress, like in salt stress (J. Liu et al., 2020), while *AtMAPK10* is related to cell division and development. A recent study found that *AtMAPK10* negatively regulates seed growth by inhibiting the transcriptional activity of *WRKY10* during endosperm development in *A. thaliana* (Xi et al., 2021). Therefore, group A may play similar regulatory roles in peanuts. Group B includes eight *AhMAPKs*, five *IpMAPKs*, five *DuMAPKs*, and *Arabidopsis AtMAPK4/5/11/12/13*. *AtMAPK4/11* are key players in the formation of the cell plate during cytokinesis (Kosetsu et al., 2010), whereas *AtMAPK12* participates in regulating auxin signaling (Lee et al., 2009). We speculate that group B MAPKs may be involved in peanut growth/development processes. The four *AhMAPK*s, two *IpMAPKs*, and two *DuMAPKs* were classed into group C, which closely relates to *AtMAPK1/2/7/14* that were reported in their response to ABA (de Zelicourt et al., 2016). Thus, this group of MAPKs may have similar functions with group B in peanut development. Group D is the largest MAPK subfamily, containing 14 *AhMAPKs*, seven *IpMAPKs*, and seven *DuMAPKs*. Members in this group are highly homologous to *Arabidopsis AtMAPK8/9/15/16/18/19/20*, which are characterized by the presence of the TDY motif in their T‐loop and an extended C‐terminal region, compared to other MAPK groups (A, B, and C). In conclusion, the peanut MAPK family represents high diversity, with different subfamily members that potentially play distinct regulatory roles in plant growth, development, and stress responses. While this study provides a foundation for understanding peanut MAPKs, further research, including gene modification through knockout or overexpression, is crucial to definitively determine the specific functions of individual MAPK family members in peanut.

Cis‐acting elements play a crucial role in governing the spatial‐temporal patterns of gene expression by serving as the binding sites where transcription factors can bind and implement their regulatory effects (Li et al., 2020). The cis‐element analysis unveils the putative cis‐regulatory landscape governing MAPK gene expression in peanut. In our results, we identified important stress/phytohormone‐responsive cis‐elements like MYB, MYC, ABRE, and ERE motifs. These elements suggest potential roles for the MAPK genes in mediating plant responses to various environmental stimuli. The prevalence of conserved stress‐responsive elements emphasizes the importance of MAPKs in stress adaptation, while the identification of hormone‐ and development‐related motifs expands our understanding of their potential regulatory mechanisms. These findings help us for future investigations into the intricate transcriptional networks orchestrating MAPK activity in response to environmental stimuli and developmental programs in peanut.

Our expression analysis revealed that *Ah_At_MAPK4* and *Ah_Bt_MAPK4* are significantly upregulated in response to drought stress, with higher induction in drought‐tolerant peanut genotypes compared to drought‐susceptible ones. Interestingly, our previous phylogenetic analysis placed these two homologous genes in group A, clustering with *AtMAPK3*, *AtMAPK6*, and *AtMAPK10* from *Arabidopsis*. In *Arabidopsis*, group A MAPKs have been extensively characterized for their critical roles in abiotic stress responses (Ichimura et al., 2002; Taj et al., 2010). Particularly, *AtMAPK3* and *AtMAPK6* function as positive regulators in response to various environmental stresses, including drought, salt, cold, and oxidative stress (J. Liu et al., 2020; Moustafa et al., 2014). The significant drought‐induced expression of *Ah_At_MAPK4* and *Ah_Bt_MAPK4* observed in our study aligns with the known functions of their *Arabidopsis* orthologs, suggesting evolutionary conservation of MAPK signaling in drought response across plant species. The substantially higher upregulation of these genes in drought‐tolerant genotypes (5.61–7.17‐fold) compared to drought‐susceptible genotypes (2.57‐ to 3.38‐fold) further indicates their potential involvement in enhanced drought tolerance mechanisms. This differential expression pattern might be attributed to more efficient activation of MAPK cascades in tolerant genotypes, potentially leading to improved regulation of downstream drought‐responsive genes and metabolism. To explore this hypothesis further, we employed WGCNA to identify co‐expression networks associated with these MAPK genes. Both *Ah_At_MAPK4* and *Ah_Bt_MAPK4* were assigned to the turquoise module, which showed the strongest correlation with drought tolerance traits (*r* = 0.89, *p* < 0.001). Functional enrichment analysis of this module revealed significant overrepresentation of processes central to drought adaptation, including terpene synthase activity, defense responses, and terpenoid biosynthesis pathways. Terpenoids are well‐established osmoprotectants that help maintain cellular osmotic balance during water deficit, and their enrichment in the MAPK‐associated module suggests these kinases may regulate osmoprotectant production as part of the drought response. Within this module, co‐expression network analysis identified 20 hub genes, many of which were annotated with calmodulin binding activity. This finding highlights the potential importance of calcium‐dependent signaling, a well‐established upstream activator of MAPK cascades, in orchestrating drought‐responsive transcriptional programs. Moreover, enrichment of diterpenoid and gibberellin biosynthesis pathways among hub genes suggests that MAPK activation may feed into secondary metabolism and hormone‐mediated growth regulation during stress conditions. Consistently, both proteins exhibited highly conserved kinase domains with canonical ATP‐binding and activation motifs, consistent with the structural architecture reported for group A MAPKs in *Arabidopsis*. In conclusion, our findings establish *Ah_At_MAPK4* and *Ah_Bt_MAPK4* as central regulators of drought tolerance in peanut, with conserved structural features that support their catalytic activity and identify them as promising targets for functional validation and genetic improvement. While our integrated multi‐omics approach provides compelling evidence for the drought‐responsive function of *Ah_At_MAPK4* and *Ah_Bt_MAPK4*, we acknowledge a limitation of the current study is the lack of quantitative reverse transcription polymerase chain reaction (qRT‐PCR) validation for these expression patterns. Future studies should validate these findings through qRT‐PCR and conduct functional characterization via gene knockout or overexpression experiments. Additionally, investigating upstream regulatory mechanisms, including miRNA‐mediated post‐transcriptional regulation, would provide a more comprehensive understanding of MAPK signaling in peanut drought responses.

## 5 CONCLUSION

In summary, genome‐wide analysis of MAPK gene families was first conducted in peanut. We identified 30 MAPK genes in *A. hypogaea*, 16 MAPK genes in *A. duranensis*, and 15 MAPK genes in *A. ipaensis*. Phylogenetic analysis clustered all the identified MAPK genes into four distinct groups, which is consistent with the previous studies. RNA‐seq expression profiling under drought stress conditions demonstrated that *Ah_At_MAPK4* and *Ah_Bt_MAPK4* were significantly upregulated in response to drought stress, with substantially higher induction in drought‐tolerant genotypes compared to drought‐susceptible ones. WGCNA further confirmed their central positions in a drought‐associated co‐expression module enriched in calcium signaling and secondary metabolic pathways, underscoring their regulatory importance in stress adaptation. In addition, AlphaFold‐based structural modeling confirmed that both proteins possess the canonical kinase architecture required for catalytic function. These findings provide valuable information for understanding the MAPK gene family in peanut and identify promising candidates for future research into their roles in stress tolerance and enhancing abiotic stress resilience in peanuts through crop improvement.

## AUTHOR CONTRIBUTIONS

**Jie Zhang**: Data curation; methodology; project administration; writing—original draft; writing—review and editing. **Qingying Meng**: Methodology; writing—review and editing. **Alvaro Sanz‐Saez**: Writing—review and editing. **Charles Chen**: Project administration; supervision; writing—review and editing.

## CONFLICT OF INTEREST STATEMENT

The authors declare no conflicts of interest.

## Supporting information




**Figure S1**. PCA of RNA‐seq transcriptome data from four peanut genotypes (C76‐16, 587, Tifrunner, and 506) under control and drought conditions. Each point represents a biological replicate colored by genotype and shaped by treatment (control or drought)

**Figure S2**. Selection of the soft‐thresholding power (β) for gene co‐expression network construction

**Table S1**. MAPK_Kaks

**Table S2**. cis‐acting element analysis of MAPK genes in peanut

**Tables S3–S5**. Genes assigned to the turquoise, blue, and grey modules identified by WGCNA.

Supporting Information

Supporting Information

**Table S6**. GO and KEGG pathway enrichment results for genes in the turquoise co‐expression module identified by WGCNA

**Table S7**. GO and KEGG pathway enrichment results for hubs

## Data Availability

The genome and protein sequences of peanut species (*Arachis hypogaea*, *Arachis duranensis*, and *Arachis ipaensis*) used in this study were obtained from PeanutBase (https://www.peanutbase.org/). The specific cultivated peanut reference genome was *A. hypogaea* cv. Tifrunner, assembly Tifrunner.gnm2.J5K5. The *Arabidopsis thaliana* MAPK protein sequences were obtained from TAIR (https://www.arabidopsis.org/). The protein kinase domain (PF00069) was downloaded from the Pfam database (https://www.ebi.ac.uk/interpro/entry/pfam/). The RNA‐seq data used for expression analysis under drought stress conditions are publicly available in the NCBI Sequence Read Archive (SRA) database under the accession number PRJNA687542. All other data generated during this study are included in the manuscript and supplementary materials.
