# Supplementary material for: Genome‐wide identification and expression analysis reveals the drought‐response MAPK genes in peanut (Arachis hypogaea L.)
Source: Plant Genome. 2025 Dec 22;18(4):e70166. doi: 10.1002/tpg2.70166 (PMC12723341; doi:10.1002/tpg2.70166)
Supplement: Supplementary file 1 [file TPG2-18-e70166-s002.pdf]

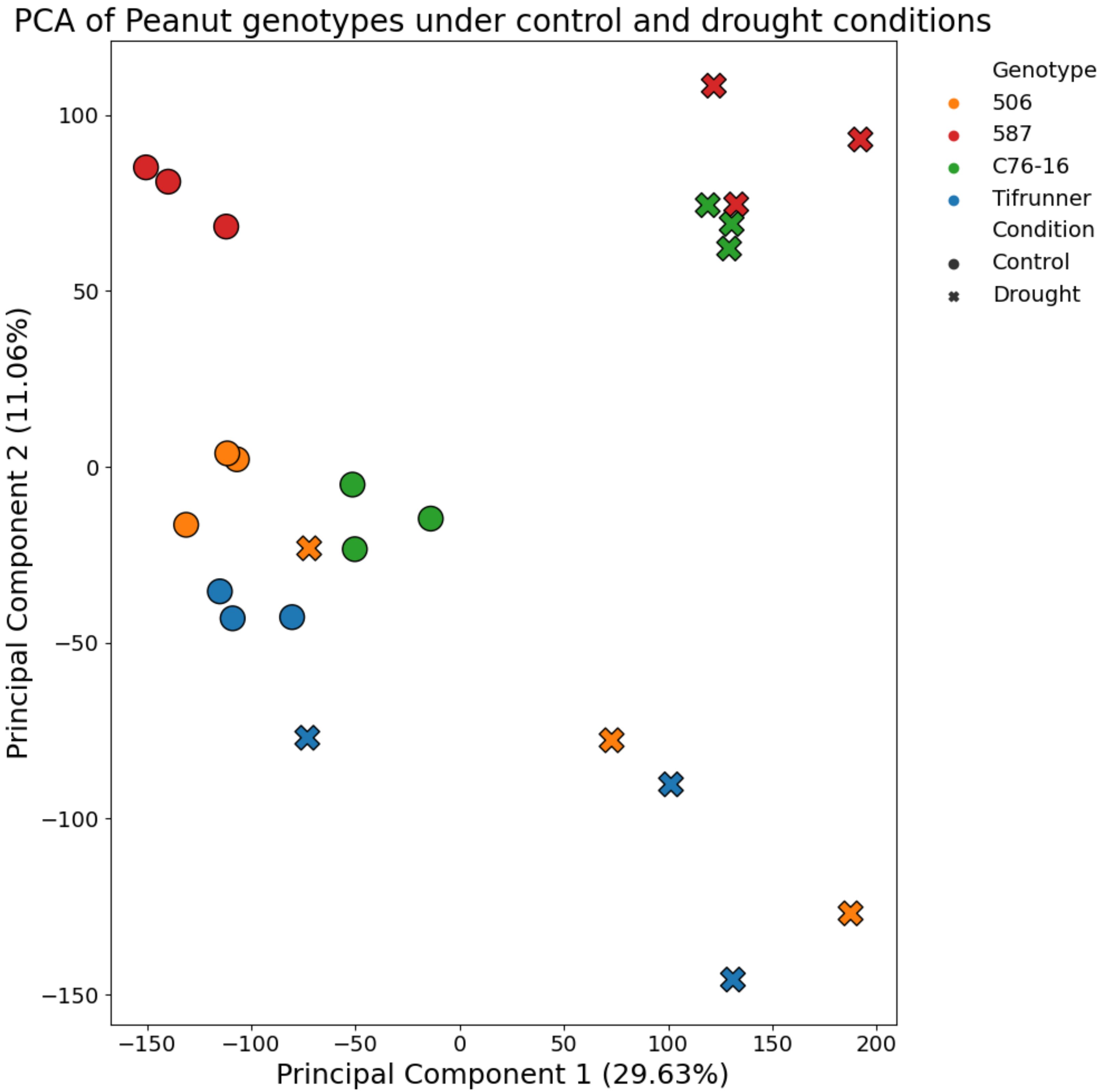
PCA of Peanut genotypes under control and drought conditions
Principal Component 1 (29.63%)
Principal Component 2 (11.06%)
−150
−100
−50
0
50
100
150
200
Genotype
506
587
C76-16
Tifrunner
Condition
Control
Drought
