# Supplementary material for: Genome‐wide identification and expression analysis reveals the drought‐response MAPK genes in peanut (Arachis hypogaea L.)
Source: Plant Genome. 2025 Dec 22;18(4):e70166. doi: 10.1002/tpg2.70166 (PMC12723341; doi:10.1002/tpg2.70166)
Supplement: Supplementary file 2 [file TPG2-18-e70166-s009.pdf]

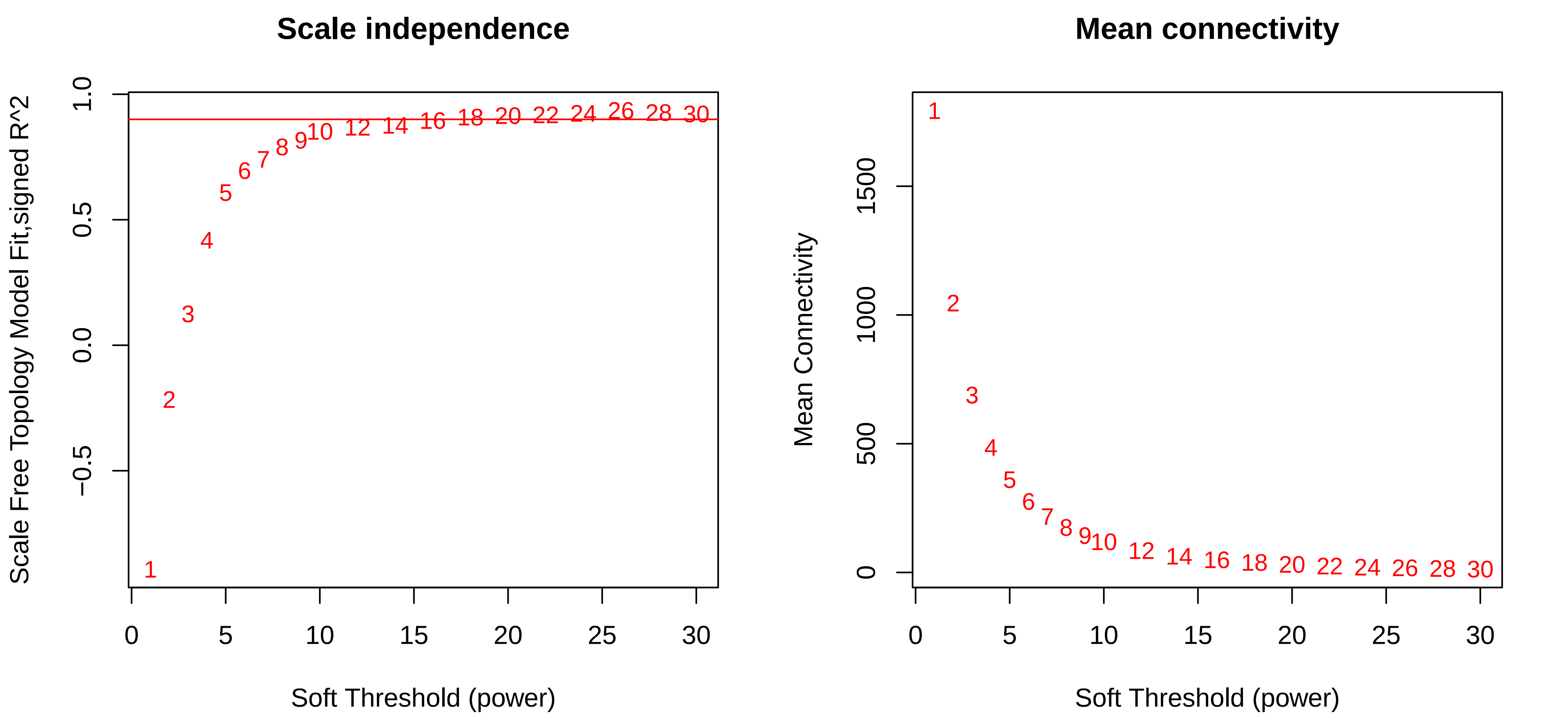
Scale independence
Scale Free Topology Model Fit,signed R^2
Soft Threshold (power)
Mean connectivity
Mean Connectivity
Soft Threshold (power)
