# Supplementary figures and images for: Genome‐wide identification and expression analysis reveals the drought‐response MAPK genes in peanut (Arachis hypogaea L.)
Source: Plant Genome. 2025 Dec 22;18(4):e70166. doi: 10.1002/tpg2.70166 (PMC12723341; doi:10.1002/tpg2.70166)

PCA of Peanut genotypes under control and drought conditions

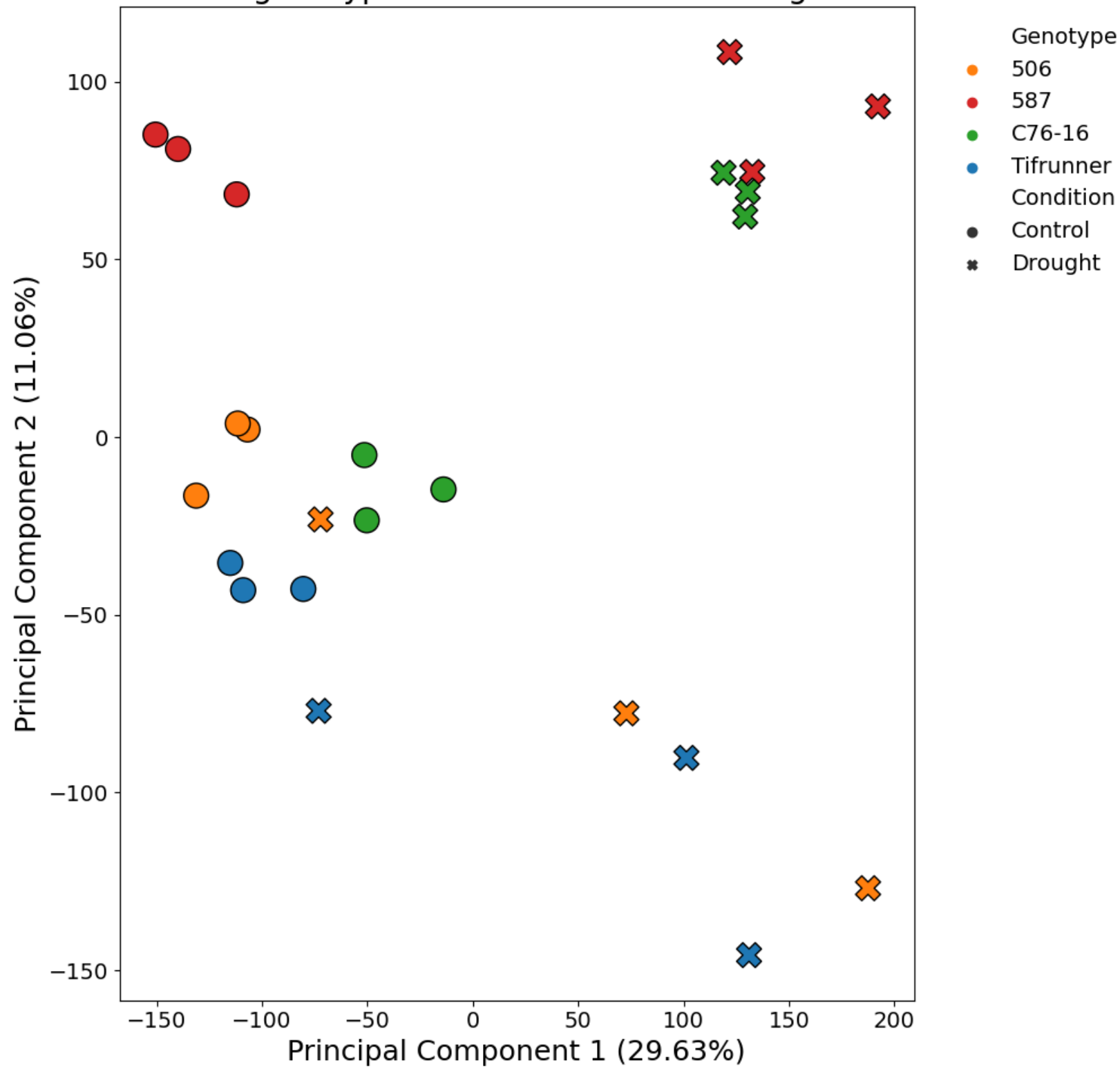

Supplement: Supplementary file 1 — Figure S1. PCA of RNA‐seq transcriptome data from four peanut genotypes (C76‐16, 587, Tifrunner, and 506) under control and drought conditions. Each point represents a biological replicate colored by genotype and shaped by treatment (control or drought) [file TPG2-18-e70166-s002.pdf]

### Scale independence

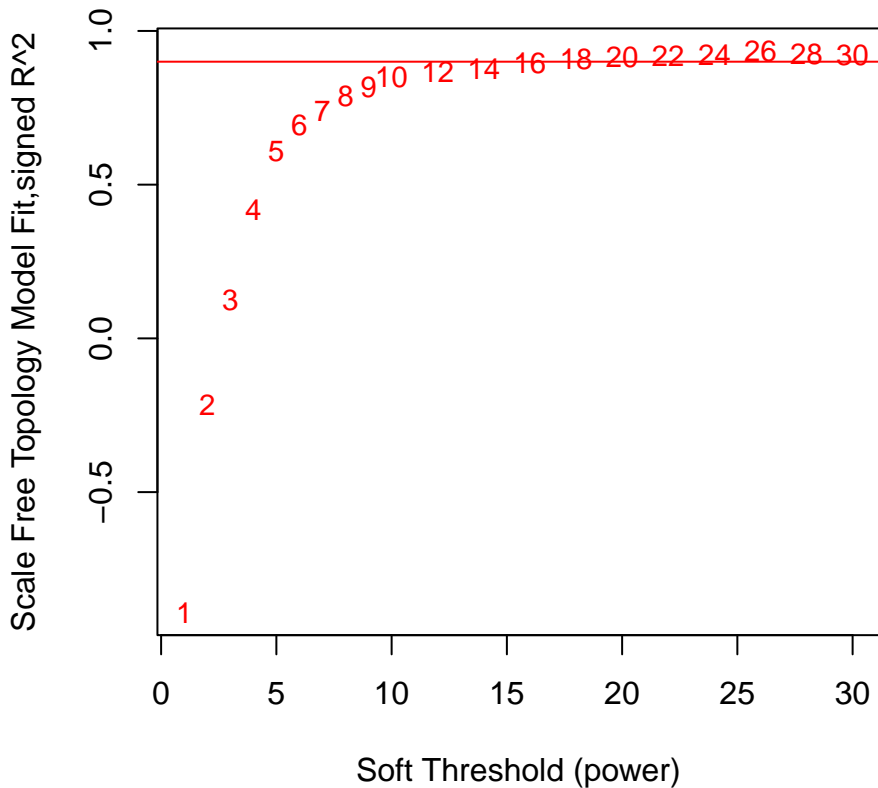

### Mean connectivity

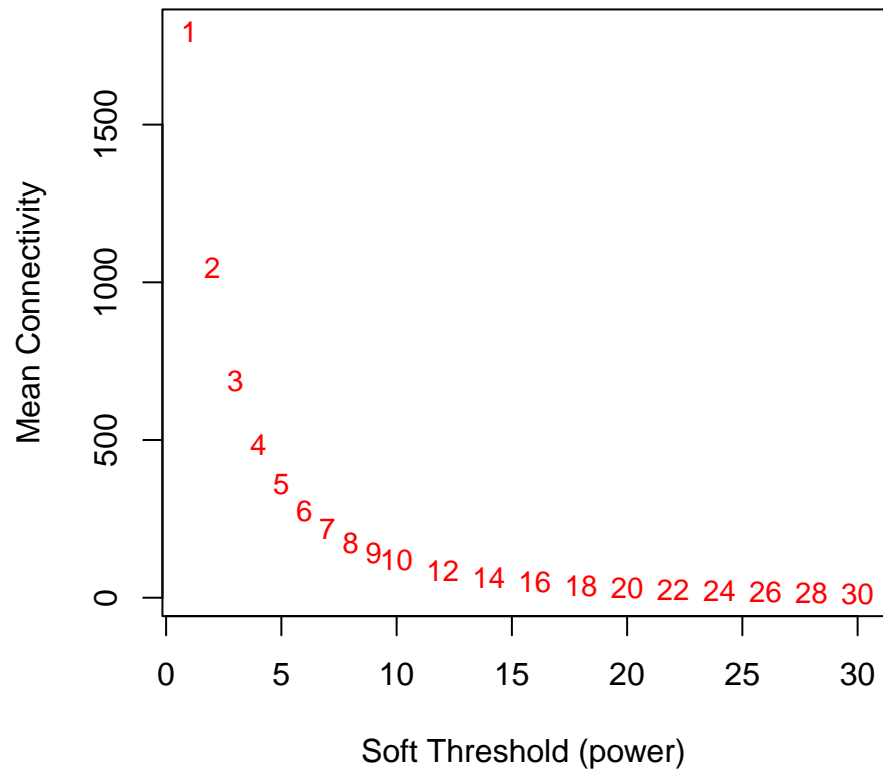

Supplement: Supplementary file 2 — Figure S2. Selection of the soft‐thresholding power (β) for gene co‐expression network construction [file TPG2-18-e70166-s009.pdf]
